# Structure of TBC1D23 N-terminus reveals a novel role for rhodanese domain

**DOI:** 10.1371/journal.pbio.3000746

**Published:** 2020-05-26

**Authors:** Dingdong Liu, Fan Yang, Zhe Liu, Jinrui Wang, Wenjie Huang, Wentong Meng, Daniel D. Billadeau, Qingxiang Sun, Xianming Mo, Da Jia

**Affiliations:** 1 Department of Pediatric Surgery and Laboratory of Stem Cell Biology, State Key Laboratory of Biotherapy, West China Hospital, Sichuan University, Chengdu, China; 2 Key Laboratory of Birth Defects and Related Diseases of Women and Children, Department of Paediatrics, West China Second University Hospital, Sichuan University, Chengdu, China; 3 Division of Oncology Research and Schulze Center for Novel Therapeutics, Mayo Clinic, Rochester, Minnesota, United States of America; National Cancer Institute, UNITED STATES

## Abstract

Members of the Tre2-Bub2-Cdc16 (TBC) family often function to regulate membrane trafficking and to control signaling transductions pathways. As a member of the TBC family, TBC1D23 is critical for endosome-to-Golgi cargo trafficking by serving as a bridge between Golgi-bound golgin-97/245 and the WASH/FAM21 complex on endosomal vesicles. However, the exact mechanisms by which TBC1D23 regulates cargo transport are poorly understood. Here, we present the crystal structure of the N-terminus of TBC1D23 (D23^N^), which consists of both the TBC and rhodanese domains. We show that the rhodanese domain is unlikely to be an active sulfurtransferase or phosphatase, despite containing a putative catalytic site. Instead, it packs against the TBC domain and forms part of the platform to interact with golgin-97/245. Using the zebrafish model, we show that impacting golgin-97/245-binding, but not the putative catalytic site, impairs neuronal growth and brain development. Altogether, our studies provide structural and functional insights into an essential protein that is required for organelle-specific trafficking and brain development.

## Abstract 2

The TBC1D23 protein functions in membrane trafficking and is critical for human neuronal development. The crystal structure of the N-terminus of TBC1D23 shows that its rhodanase domain is catalytically inactive and positioned close to the adjacent TBC domain, which together provide a binding interface for golgin-97/245. This interaction is essential for neuronal growth and brain development in zebrafish.

## Introduction

In eukaryotic cells, membrane vesicle trafficking involves movement of molecules (cargoes) between membrane organelles, in the form of membrane-bound microsized vesicles [1,2]. Vesicle trafficking is fundamental to many cellular and physiological processes, including signaling transduction, metabolic homeostasis, synaptic neurotransmission, endocrine secretion, et al. [1,3]. Dysfunctions in vesicle trafficking contribute to certain types of cancer, immunological disorders, diabetes, and most importantly, many kinds of neurological disorders [4,5]. The Rab family of small GTPases play a central role in vesicular trafficking and regulate many steps, including vesicle formation, vesicle movement, and membrane fusion [6–8]. Members of the Tre2-Bub2-Cdc16 (TBC) family often participate in membrane trafficking by functioning as Rab GTPase-activating proteins (GAPs) [9,10]. For instance, TBC1D5 is a GAP for both Rab7a and Rab7b, thus inactivating their activities [11–14]. TBC1d5 forms a stable complex with retromer and regulates retromer-dependent trafficking [12]. Furthermore, TBC1D5 and retromer cooperate in controlling late endosomal and lysosomal functions and signaling from lysosomes, such as the mammalian target of rapamycin (mTOR) pathway [15,16].

TBC1D23 is a recently characterized member of the TBC family and is conserved in many eukaryotic taxa [17]. Unlike many members of the TBC family, TBC1D23 lacks the catalytic Arg-Gln residues and unlikely functions as an active GAP [17,18]. In mammalian cells, TBC1D23 is localized on the trans-Golgi network (TGN) and functions as an adaptor to capture the incoming endosome-derived vesicles [17,19]. Thus, TBC1D23 mediates endosome-to-Golgi retrieval of many cargo molecules, such as cation-independent mannose-6-phosphate receptor (CI-MPR) and TGN46 [17,19]. Emphasizing the importance of TBC1D23 in development and human diseases is the findings that homozygous mutations of the TBC1D23 gene led to pontocerebellar hypoplasia (PCH) and/or intellectual disability [18,20,21]. PCH is a group of neurodevelopmental disorders characterized by impaired brain development, delayed overall development, and intellectual disability [22,23]. How dysregulation of endosome-to-Golgi trafficking, a core cell biology pathway, leads to the pathogenesis of PCH remains to be addressed.

Golgins are a large class of tethering proteins found at the Golgi [24,25]. Two members of the golgin family, golgin-97 and golgin-245, are targeted to the Golgi through the association of their C-terminal GRIP domains and the activated Arl1 GTPase localized at the TGN [26–29]. The N-terminal regions of golgin-97/245 interact with the TBC domain of TBC1D23, thus promoting the TGN localization of TBC1D23 [17]. In addition to the N-terminal TBC domain, TBC1D23 also possesses a rhodanese-like domain and a C-terminal domain that displays little sequence similarity with other proteins. A rhodanese domain often associates with either sulfurtransferase or phosphatase activity [30,31]; however, it is unclear whether the rhodanese domain of TBC1D23 possess any enzymatic activities. Furthermore, TBC1D23 also associates with a poorly characterized trimeric complex, consisting of WDR11, FAM91A, and C17orf75 [17,19,32]. We have recently determined the crystal structure of the TBC1D23 C-terminal domain and showed that it adopts a Pleckstrin homology (PH) domain fold [33]. We also demonstrated that the TBC1D23 C-terminal domain selectively binds to phosphoinositides, in particular phosphatidylinositol 4-phosphate (PtdIns(4)P), through one surface while binding the FAM21 subunit of the WASH complex on endosomal vesicles via the opposite surface [33–38].

Despite these advances, there is no structural information on the TBC and rhodanese domains of TBC1D23. The molecular mechanism underlying recognition of golgin-97/245 by TBC1D23 is unknown. Furthermore, although the rhodanese domain of TBC1D23 contains a putative active-site cysteine residue (C399), which is required for enzymatic activity of a rhodanese domain, it is unclear whether it functions as an active enzyme. We set to address these questions by utilizing multidisciplinary approaches, including biochemical, structural, cellular, and zebrafish studies. Here, we present the crystal structure of the N-terminus of TBC1D23 (D23^N^), which encompasses both the TBC and rhodanese domains. The two domains tightly pack against each other, forming a platform to interact with golgin-97/245. Both structural and biochemical analysis reveal that the rhodanese domain is unlikely to be an active sulfurtransferase or phosphatase, with C399 deeply buried in the structure. Consistent with biochemical and cellular observations, we demonstrate that golgin-97/245-interacting residues, but not C399, are essential for neuronal growth and brain development in zebrafish.

## Results

### Crystal structure of TBC1D23 N-terminus

Previous studies have identified the TBC domain of TBC1D23 as the key binding module for golgin-97/245 but provided little insight into molecular functions for the rhodanese domain of TBC1D23 [17]. We set out to address three questions: (1) the overall structure of the TBC and rhodanese domains; (2) molecular functions of the rhodanese domain; (3) molecular mechanism of recognition of golgin-97/245 by TBC1D23. After screening many different expression constructs, we were able to obtain diffraction-quality crystals of the TBC1D23 N-terminal region (D23^N^) consisting of residues 1–460 and encompassing both the TBC and rhodanese domains. The crystal structure of D23^N^ was determined by selenium single-wavelength anomalous diffraction (Se-SAD) and refined to a resolution of 2.5 Å (S1 Table).

**Fig 1:**
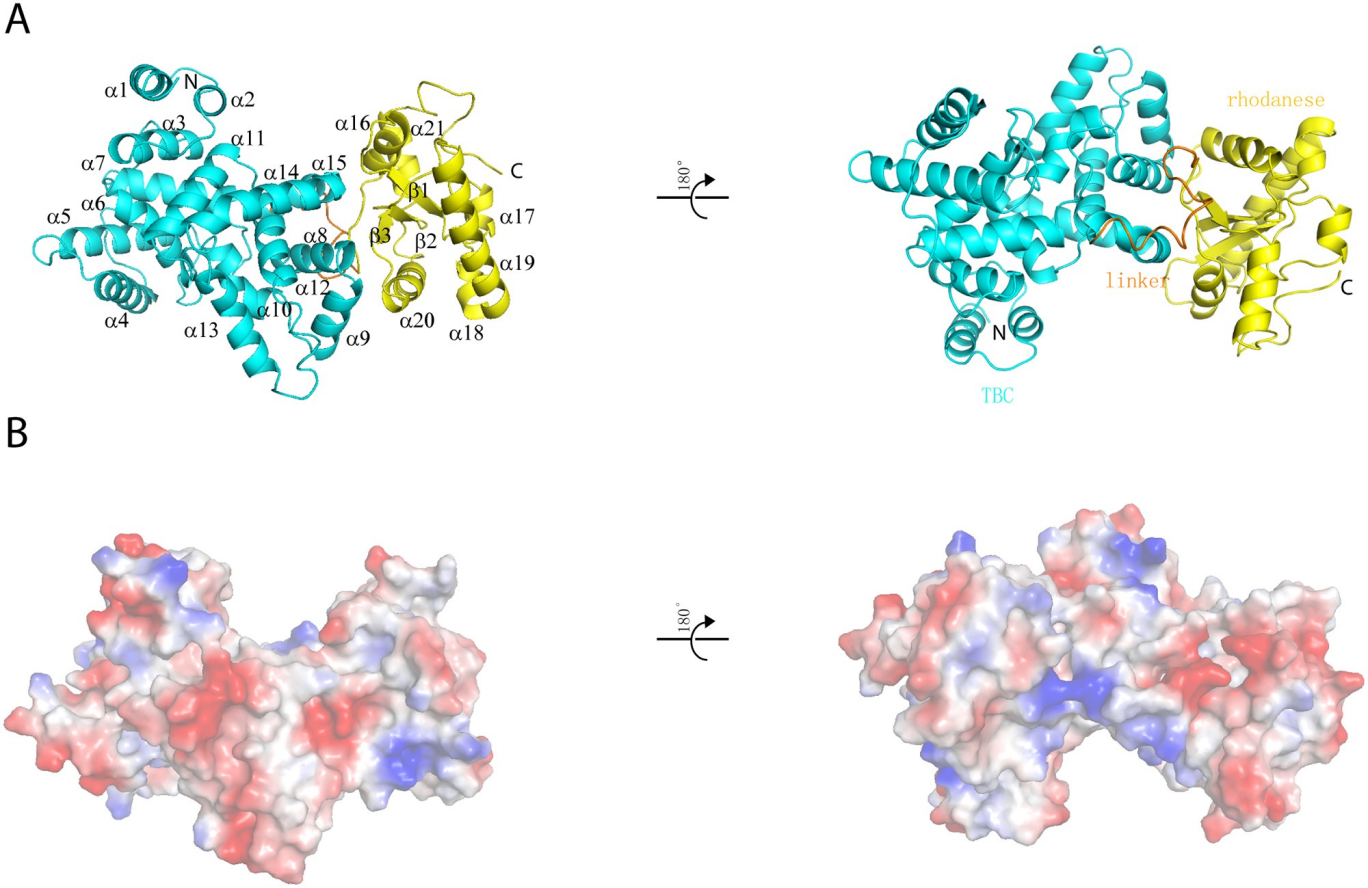
Crystal structure of D23^N^. (A) Ribbon diagrams of D23^N^, shown in two orientations rotated 180° with respect to each other. Cyan: TBC domain; gold: linker; yellow: rhodanese domain. (B) Electrostatic surface potential map of D23^N^, shown in two orientations rotated 180° with respect to each other. Blue: positive; red: negative; white: neutral. The molecules are in the same orientations as those above them in (A). D23^N^, N-terminus of TBC1D23; TBC, Tre2-Bub2-Cdc16.

Each crystallographic asymmetric unit contains one D23^N^ molecule, and the final model contains residues 14–298 (TBC domain, cyan) and residues 316–460 (rhodanese domain, yellow), connected by a short loop (amino acids [aa] 297–315; gold color), which adopts a 3_10_-helix within its N-terminus (Fig 1A). The first 13 residues of TBC1D23 are not visible in the electron density, suggesting that its conformation may be flexible. The TBC and rhodanese domains tightly pack against each other and bury a total solvent-accessible surface area of 1,350 Å^2^ (Fig 1 and S1 Fig). The two domains together adopt a V-shaped structure, with the TBC domain forming the base and one arm and the rhodanese domain the other arm (Fig 1).

The interaction between the TBC and rhodanese domains involves multiple hydrogen bonds and hydrophobic interactions (S1A Fig). For instance, S289 from the TBC domain forms multiple hydrogen bonds with the side chains of S321 and E324 from the rhodanese domain. Y177 displays hydrogen bond interactions with the side chain of H397 and the amide of Y426. In addition to hydrogen bonds, various residues from the TBC and rhodanese domains engage through van der Waals interactions. The phenyl group of Y177 stacks between the side chains of L318 and Y426 (S1A Fig). Furthermore, the side chain of L318 inserts into a hydrophobic pocket formed by residues L173, Y177, P287, and F290 (S1A Fig). The intricate interactions between the two domains suggest that the two domains may stabilize each other and function together. Indeed, when we attempted to produce proteins encompassing only the rhodanese domain, all of our expression constructs resulted in insoluble proteins or soluble aggregations. Finally, many residues involved in the interaction between the two domains are conserved throughout evolution, suggesting that TBC1D23 from other organisms likely adopt a similar configuration (S2 Fig).

Similar to other TBC-containing proteins, the TBC domain of TBC1D23 only compromises α-helices, which can be further divided into two subdomains: the amino-terminal domains containing α1–α7 and a carboxy-terminal subdomain spanning α8–α16 (Fig 1A and 1B) [39]. Structural comparison using Dali [40] showed that the TBC domain of TBC1D23 displays significant similarity with that of Gyp1p (DALI Z-score = 23), a GAP for Rab33 (S1B Fig) [39]. The most noticeable difference between two domains is that helix α4 in Gyp1p is missing in TBC1D23 (S1B Fig). Despite these overall similarities, two structures show clear differences in details, indicated by a root-mean-square deviation (rmsd) value of 2.8 Å for 263 Cα atoms. Previous studies have identified two signature motifs critical for the GAP activity, the IxxDxxR and YxQ motifs [39]. Our structure readily explained why the TBC domain of TBC1D23 is not an active GAP for Rab GTPases. First, the arginine residue in the IxxDxxR motif is replaced by glutamine in TBC1D23 (Q88), and the distance of Cα atoms between Q88 of TBC1D23 and R343 of Gyp1p is more than 5.9 Å (S1C Fig). Second, a threonine (T124) residue substitutes the glutamine residue in the YxQ motif (S1C Fig). T124 is unable to mediate bipartite hydrogen-bonding interactions with a fluoride ion and water, unlike the glutamine residue [39]. Consistent with our structural analysis, both full-length (FL) TBC1D23 [18] and the TBC domain alone did not display any obvious GAP activity toward a panel of Rab GTPases (S3 Fig).

**Fig 2:**
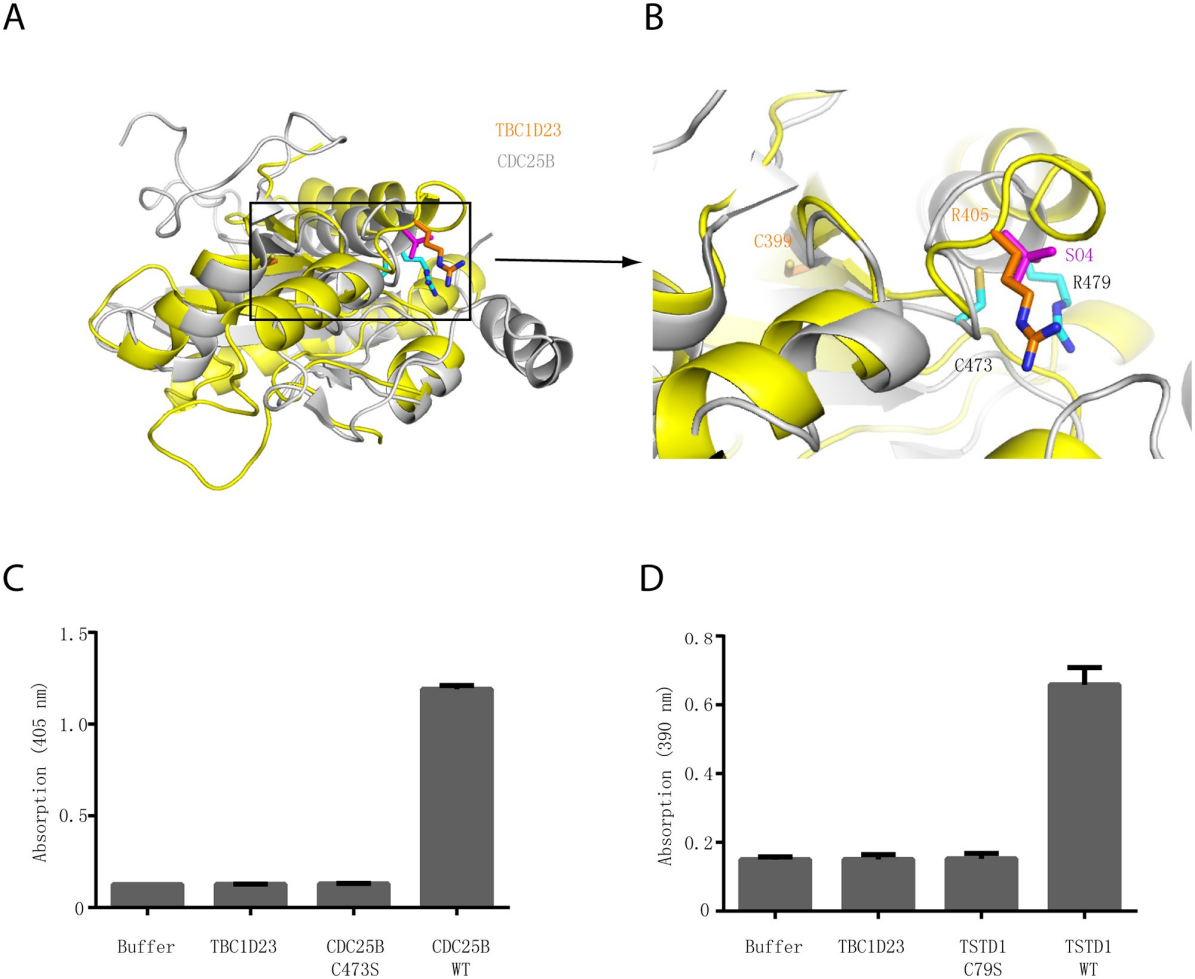
The rhodanese domain of TBC1D23 is unlikely to possess phosphatase or sulfurtransferase activity. (A) Structural comparison of the crystal structures of TBC1D23 rhodanese domain (yellow) and of CDC25B (gray) (PDB ID: 1qbo). (B) Comparison of the activity site of CDC25B and the corresponding region of TBC1D23. Two essential residues (C473 and R479) from the catalytic CX_5_R motif of CDC25B are shown and labeled with black font. The corresponding residues (C399 and R405) in TBC1D23 are labeled in gold font. The SO_4_^2+^ found in the catalytic site of CDC25B is colored in magenta. (C) The phosphatase activity of CDC25B WT, C473S, and TBC1D23 (aa1–460). pNPP was utilized as substrates. The reaction was carried out at 25 °C, and absorbance at 405 nm was monitored. Data are from three replicate experiments (mean ± S.D.), and the numerical data are included in S1 Data. (D) The sulfurtransferase activity of TSTD1 WT, C79S, and TBC1D23 (aa1–460). (E) H_2_S formed by the reaction of thiosulfate and GSH was determined in the lead sulfide assay, and absorbance at 390 nm was monitored. Data are from three replicate experiments (mean ± S.D.), and the numerical data are included in S1 Data. aa, amino acid; GSH, glutathione; PDB, Protein Data Bank; pNPP, disodium 4-nitrophenyl phosphate; TSTD1, thiosulfate sulfurtransferase like domain containing 1; WT, wild type.

The rhodanese domain of TBC1D23 adopts α/β topology typical of a single rhodanese domain (Fig 2A). The domain contains a central parallel β-sheet composed of three β strands, flanked by three helices on each side. DALI search [40] also reveals that the rhodanese domain of TBC1D23 displayed significant structural similarity with other rhodanese domains, such as those from protein phosphatase CDC25B (Z-score = 9.3) [41], and human thiosulfate sulfurtransferase like domain containing 1 (TSTD1) (Z-score = 9.0) [42]. Comparisons between TBC1D23 and these rhodanese domain-containing proteins will be further discussed in the following section.

### TBC1D23 is unlikely to be an active phosphatase or sulfurtransferase

The rhodanese domain is a small domain (about 120 residues) defined by the sequence and structural similarity and can be found within all three major evolutionary phyla [30]. Proteins with a rhodanese domain could be active enzymes possessing sulfurtransferase or phosphatase activity. Catalytic rhodanese modules often possess a Cx_4_R or Cx_5_R motif, which forms the catalytic pocket [30]. TBC1D23 also harbors a putative enzymatic motif (C399x_5_R405) within its rhodanese domain; however, it is unknown whether this domain possesses any enzymatic activities [43].

In the crystal structure of CDC25B, the signature Cx_5_R motif is within a loop connecting the central β5 strand of the β-sheet and helix α4 (Fig 2A and 2B) [41]. Located at the protein surface, this active-site loop forms a cradle-like conformation, which is mainly positively charged residues. The geometry of the pocket together with the positively charged surroundings allows it to bind substrates containing a phosphate group. The C473 residue is located at the bottom of the pocket and is essential for catalysis by acting as a nucleophile [41]. In contrast with CDC25B, the putative active-site cysteine residue C399 is localized in the middle of β2 strand of the β-sheet, and its side chain is solvent-inaccessible (Fig 2A and 2B). Similarly, although R405 is localized in the loop connecting a β strand and a helix, it is far away from its equivalent residue in CDC25B, R479, with the distance of Cα atoms of two residues being more than 10 Å (Fig 2A and 2B). Thus, our structural analysis indicates that the rhodanese domain of TBC1D23 is unlikely to be an active phosphatase or sulfurtransferase.

Since the rhodanese domain of TBC1D23 displays high structural similarity with that of CDC25B (Dali Z-score = 9.3) or TSTD1 (Z-score = 9.0), we chose substrates and used the experimental conditions that have been established for these two enzymes [41,42]. To assess whether TBC1D23 possesses any phosphatase activity, we performed in vitro phosphatase assay and utilized CDC25B as a positive control (Fig 2C). CDC25B wild type (WT) effectively removed the phosphate group from disodium 4-nitrophenyl phosphate (pNPP), forming a product with strong absorption at 405 nm. Mutation of the catalytic cysteine in CDC25B, C473S, completely abolished its phosphatase activity. In contrast with CDC25B, TBC1D23 did not display any phosphatase activity (Fig 2C). Next, we tested whether TBC1D23 is an active sulfurtransferase, like TSTD1 (Fig 2D). In accordance with previous studies, TSTD1 WT effectively converted thiosulfate into hydrogen sulfide, which eventually formed lead sulfide that has absorption at 390 nm [42]. Substitution of the active-site cysteine in TSTD1, C79S, fully abrogated its sulfurtransferase activity, indicated by the absorption at 390 nm. Under the same experimental condition, we did not observe any sulfurtransferase activity for TBC1D23 (Fig 2D). Similarly, we detected robust sulfurtransferase activity for TSTD1 WT, but not for TSTD1 C79S or TBC1D23, when different substrates were used in the assay (S4 Fig). Altogether, our structural and biochemical analysis suggest that the rhodanese domain of TBC1D23 likely functions though means other than being an active phosphatase or sulfurtransferase.

### The rhodanese domain is required for the subcellular localization and functions of TBC1D23, independent of residues 399/405

**Fig 3:**
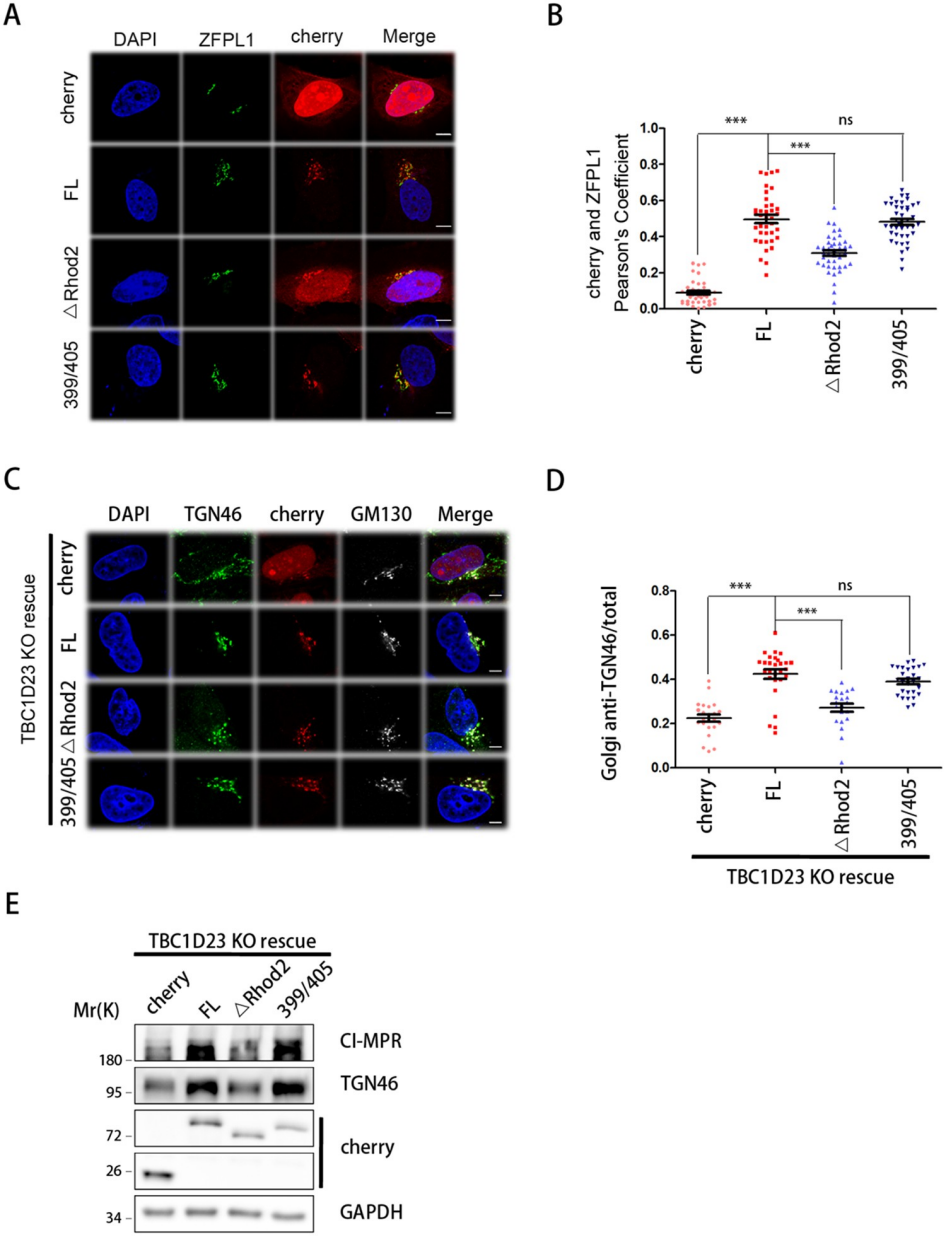
The rhodanese domain, but not residues 399/405, is required for the subcellular localization and functions of TBC1D23. (A) HeLa cells were transfected with mCherry or mCherry-TBC1D23 FL (“FL”), ΔRhod2 (deleting aa331–460), or C399S/R405A (399/405) and then fixed and labeled with anti-ZFPL1 (Golgi marker, green) antibody. Scale bar: 10 μm. (B) Quantitation of mCherry colocalization with ZFPL1 in cells as treated in (A). Each dot represents Pearson’s correlation coefficients from one cell. *P* values were calculated using one-way ANOVA, post hoc Tukey’s test. ***P < 0.001. Experiments were triplicated, and the numerical data are included in S1 Data. (C) TBC1D23-KO HeLa cells were transfected with mCherry or FL, ΔRhod2, or 399/405 and then fixed and labeled with anti-TGN46 (green) and GM130 (Golgi marker, white) antibodies. TGN46 is recycled between endosomes and Golgi in a TBC1D23-dependent manner. Scale bar: 10 μm. (D) Quantitation of Golgi-localized TGN46 over its total amount in cells treated as in (C). Each dot represents results from one cell. *P* values were calculated using one-way ANOVA, post hoc Tukey’s test. ***P < 0.001. Experiments were triplicated, and the numerical data are included in S1 Data. (E) Immunoblot of whole-cell extracts showing the total protein level of CI-MPR and TGN46 in cells treated in (C). aa, amino acid; CI-MPR, cation-independent mannose-6-phosphate receptor; FL, full-length; GAPDH, glyceraldehyde 3-phosophate dehydrogenase; KO, knockout; ns, not significant; TGN, trans-Golgi network; ZFPL1, zinc finger protein like 1.

Previous studies have provided definitive functions for the TBC and the C-terminal PH domain of TBC1D23 [17,33]; in contrast, the molecular functions of the rhodanese domain remained obscure. Since the TBC domain of TBC1D23 is catalytically inactive, the rhodanese domain unlikely functions to suppress its GAP activity (S3 Fig). In order to dissect the functions of the rhodanese domain of TBC1D23, we first determined its contribution to protein subcellular localization. FL TBC1D23 displayed strong colocalization with the Golgi marker zinc finger protein like 1 (ZFPL1), consistent with published studies (Fig 3A and 3B) [17,33]. Deletion of the rhodanese domain (ΔRhod2) decreased the accumulation of TBC1D23 on the Golgi (Fig 3A and 3B). In contrast, the 399/405 mutant (C399S/R405A), which replaces two “key residues” within the putative catalytic site of the rhodanese domain, did not alter the subcellular localization of TBC1D23 (Fig 3A and 3B).

To determine whether the rhodanese domain contributes to the cellular functions of TBC1D23, we chose TGN46 as a model cargo, which recycles between endosomes and TGN in a TBC1D23-dependent manner [17,33]. Consistent with previous studies [17,33], deletion of TBC1D23 resulted in a loss of TGN46 Golgi accumulation and increased lysosomal degradation of TGN46 protein (Fig 3C–3E). In this background, transduction of TBC1D23 FL, but not ΔRhod2, effectively restored the localization of TGN46 to the Golgi, determined by quantitative immunofluorescence, and the TGN46 protein level, detected by immunoblotting (Fig 3C–3E). The 399/405 mutant did not impact either the subcellular localization of TGN46, or its total protein level, consistent with the notion that it may not function as an active enzyme (Fig 3C–3E). Taken together, these data indicate that the rhodanese domain contributes to the subcellular localization and cellular functions of TBC1D23, independent of its putative active site (C399/R405).

### Both TBC and rhodanese domains of TBC1D23 interacts with golgin-97 and golgin-245

**Fig 4:**
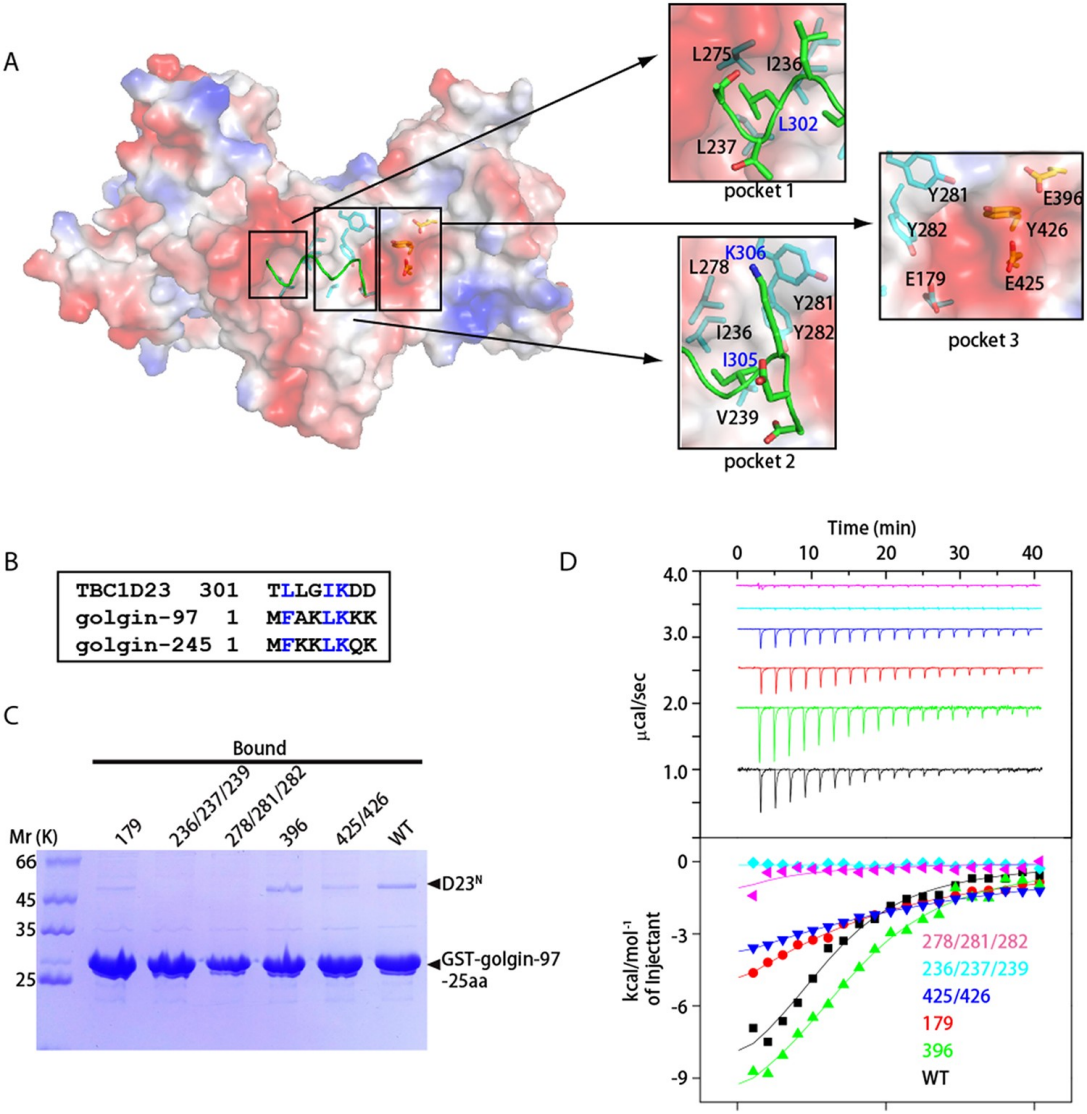
Both TBC and rhodanese domains of TBC1D23 interact with golgin-97/245. (A) Surface representation of one D23^N^ monomer (molecule 1), with a fragment from symmetry-related D23^N^ molecule shown as sticks (molecule 2, green). Molecule 1 is shown in the identical orientation to that in Fig 1B. Critical residues involved in dimerization are highlighted on the right, with residues from molecule 1 and 2 labeled in black and blue fonts, respectively. (B) Sequence alignment between residues from D23^N^-linker, N-terminus of golgin-97 and of golgin-245. Blue color indicates same or similar type of aa. (C) GST pull-down assays performed with GST-golgin-97-25aa and purified D23^N^ WT, E179K (“179”), L278A/Y281A/Y282A (“278/281/282”), I236A/I237A/V239A (“236/237/239”), E396K (“396”), or E425K/Y426A (“425/426”). After incubation with soluble protein(s), the resin was extensively washed. The resin-bound proteins were then subjected to SDS-PAGE and Coomassie Blue staining. (D) ITC experiments for the binding of golgin-97-25aa peptide with D23^N^ WT, E179K (“179”), L278A/Y281A/Y282A (“278/281/282”), I236A/I237A/V239A (“236/237/239”), E396K (“396”), or E425K/Y426A (“425/426”). Top and bottom panels show raw and integrated heat from injections, respectively. The solid curves in the bottom panel represent a fit of the integrated data to a single-site binding model. Experiments were triplicated, and the numerical data are included in S1 Data. aa, amino acid; D23^N^, N-terminus of TBC1D23; GST, glutathione S-transferase; ITC, isothermal titration calorimetry; TBC, Tre2-Bub2-Cdc16; WT, wild type.

In order to understand the mechanism of recognition of golgin-97 and golgin-245 by TBC1D23, we attempted to co-crystallize various golgin-97 peptides with D23^N^, or to soak the peptides with preformed crystals. Unfortunately, we were not able to obtain the complex structure through either approach. We suspected that the present crystallographic packing disfavored the interaction between TBC1D23 and golgin-97 or golgin-245. D23^N^ possesses several deep pockets lining the center of its surface, with pocket 1 and 2 being predominately hydrophobic and pocket 3 consisting of both aromatic and negative charged residues (Fig 4A). Interestingly, a loop connecting the TBC and rhodanese domain**s** (aa301–308) from the neighboring TBC1D23 molecule (D23^N2^) makes multiple contacts with residues forming these pockets (Fig 4A). Specifically, the side chain of L302 (D23^N2^) is deeply inserted into hydrophobic pocket formed by I236, L237, and L275. Similarly, the side chain of I305 (D23^N2^) is inserted to pocket 2, which is formed by I236, V239, and Y282. Furthermore, K306 of D23^N2^ interacts with Y281 of D23^N1^ through cation-π interaction.

Unexpectedly, sequences of the N-terminal eight residues of golgin-97 and golgin-245 display some similarity with aa301–308 of TBC1D23, which is involved in the interactions between TBC1D23 molecules within the crystal structure (Fig 4B). Residues 301–308 of TBC1D23 adopt a 3_10_-helical structure. Similarly, the N-terminal 14 residues of golgin-97 and golgin-245 are predicted to adopt an α-helical structure (S5 Fig). Previous biochemical studies have identified L2 and F5 residues of golgin-97 and golgin-245 as the most critical residues interacting with TBC1D23 [17]. These two residues resemble L302 and I305 of TBC1D23, respectively (Fig 4B). Furthermore, the K6 of golgin-97 and golgin-245 corresponds to K306 of TBC1D23 (Fig 4B). Additionally, golgin-97 and golgin-245 encompass several positive-charged residues after K6 (K7 and K8 in golgin-97, and K8 in golgin-245), which may contact the negative charged residues in pocket 3.

The above observations raise the possibility that the N-terminus of golgin-97 and golgin-245 may bind the three pockets of TBC1D23, in a manner analogous to that of aa301–308. To test this possibility, we first determined the optimal length of golgin-97 peptides required to interact with TBC1D23 using both glutathione S-transferase (GST) pull-down and isothermal titration calorimetry (ITC) assays (S6A–S6C Fig). Whereas the golgin-97 25-aa peptide robustly retained D23^N^ in the pull-down assay, the amount of bound D23^N^ was decreased when the length of the peptide was shortened to 12 or 8 aa. Consistently, ITC measurement indicated that the golgin-97 25-aa peptide bound to D23^N^ with a stoichiometry of 1:1, and with a moderate affinity (Kd ≈ 12 μM) (S6C Fig). The 12- or 8-aa peptides reduced the affinity more than 10-fold (S6C Fig). Thus, we used the 25-aa peptide in the following studies.

To test whether golgin-97 and aa301–308 of D23^N2^ binds analogously to TBC1D23, we introduced a number of mutations in residues forming the three pockets of TBC1D23 (Fig 4C and 4D). All mutant proteins likely adopted a correct folding, as they eluted from size exclusion chromatography as a monomer, similar to the WT protein. Among them, two mutant proteins (236/237/239: I236A/I237A/V239A; 278/281/282: L278A/Y281A/282A) showed the most obvious reduction and nearly abolished the interaction in the pull-down assay (Fig 4C). Two other mutants (179: E179K; 425/426: E425K/Y426A), including mutation within the rhodanese domain (425/426), showed significantly reduced affinity (Fig 4C). In contrast, the E396K mutant retained similar amount of D23^N^ as WT. Quantitative measurement using ITC revealed a similar trend: The mutants E179K and E425K/Y426A reduced the binding affinity by approximately 5-fold, and the E396K mutant slightly decreased the affinity (Kd ≈ 18 μM) (Fig 4D). The binding of both triple mutants (I236A/I237A/V239A, L278A/Y281A/282A) was too low to be determined (Fig 4D). Similar results were obtained in GST pull-down assays using golgin-245, indicating that golgin-245 binds to the same region of TBC1D23 (S6D Fig). Finally, a double mutation within the golgin-97 (K6E/K8E) significantly reduced the amount of D23^N^ protein retained on the resin, consistent with the idea that these positive residues could interact with residues from pocket 3, which is predominately negative charged (S6D Fig). Altogether, our data suggest that golgin-97 and golgin-245 likely bind to the three pockets on the surface of TBC1D23, through a manner analogous to that of residues 301–308 observed in the crystal structure.

Both TBC1D23 and golgin-97/245 are highly conserved throughout evolution (S2 Fig). To further assess whether the interaction between TBC1D23 and golgin-97/245 is conserved, we chose to study proteins from two model organisms: fly and worm. In the GST pull-down assay, both fly and worm D23^N^ proteins were retained by immobilized GST-golgin-97, but not by GST, similar to their human ortholog (S6F Fig). In contrast, triple mutants analogous to human I236A/I237A/V239A in both organisms (I223A/I224A/I226A in fly and V220A/F221A/V223A in worm) greatly reduced the interaction (S6F Fig). These data suggest that the interaction between golgin-97/245 and TBC1D23 is likely to be conserved, at least in metazoans.

### Mutation of key TBC1D23 residues impairs golgin-97/245 interaction and cargo trafficking in cells

**Fig 5:**
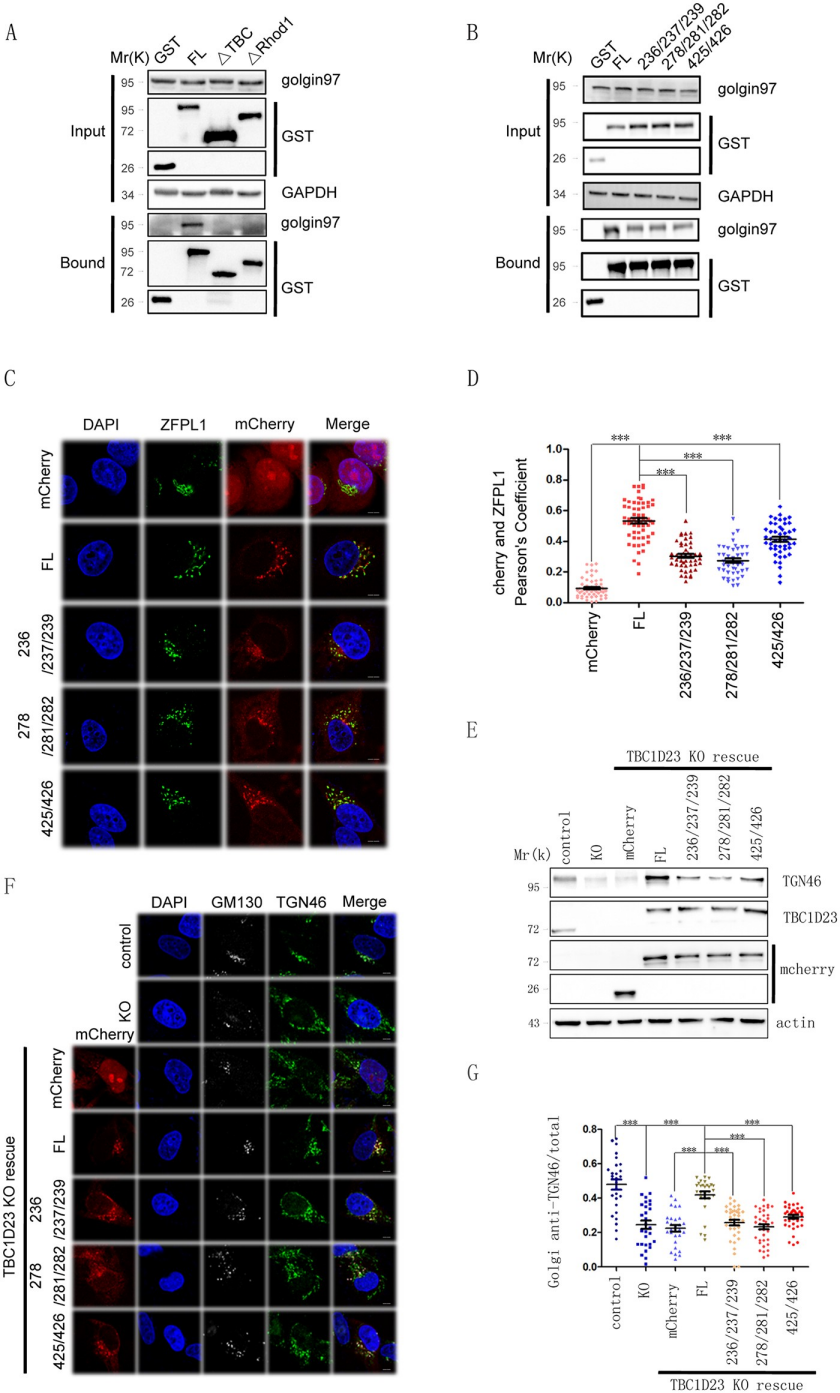
Mutations that weaken golgin-97/245-binding impair subcellular localization and functions of TBC1D23. (A) GST pull-down analyses of HEK 293T cells transfected with vectors expressing GST, GST-TBC1D23-FL (“FL”), GST-TBC1D23-ΔTBC (deleting aa1–330), or GST-TBC1D23-ΔRhod1 (deleting aa331–513). Cell lysates were precipitated with glutathione-Sepharose beads and probed with anti-GST, golgin-97, or GAPDH (control) antibodies. (B) GST pull-down analyses of HEK 293T cells transfected with vectors expressing GST, GST-TBC1D23-FL (“FL”), TBC1D23-L278A/Y281A/Y282A (“278/281/282”), TBC1D23-I236A/I237A/V239A (“236/237/239”), or TBC1D23-E425K/Y426A (“425/426”). Cell lysates were precipitated with glutathione-Sepharose beads and probed with anti-GST, golgin-97, or GAPDH (control) antibodies. (C) HeLa cells were transfected with mCherry, or mCherry-TBC1D23 FL (“FL”), TBC1D23-L278A/Y281A/Y282A (“278/281/282”), TBC1D23-I236A/I237A/V239A (“236/237/239”), or TBC1D23-E425K/Y426A (“425/426”) and then fixed and labeled with anti-ZFPL1 (blue) antibody. Scale bar: 10 μm. (D) Quantitation of mCherry colocalization with ZFPL1 (Golgi marker) in cells as treated in (C). Each dot represents Pearson’s correlation coefficients from one cell. *P* values were calculated using one-way ANOVA, post hoc Tukey’s test. ***P < 0.001. Experiments were triplicated, and the numerical data are included in S1 Data. (E) Immunoblot of whole-cell extracts of parental HeLa Cells (control), TBC1D23 KO cells (“KO”), TBC1D23 KO cells transfected with vectors expressing mCherry, mCherry-TBC1D23-FL (“FL”), TBC1D23-L278A/Y281A/Y282A (“278/281/282”), TBC1D23-I236A/I237A/V239A (“236/237/239”), or TBC1D23-E425K/Y426A (“425/426”). Cell lysates were probed with anti-cherry, TBC1D23, TGN46, or actin (control) antibodies. (F) Confocal immunofluorescence of TBC1D23 KO HeLa cells transfected with vectors expressing mCherry, mCherry-TBC1D23-FL (“FL”), TBC1D23-L278A/Y281A/Y282A (“278/281/282”), TBC1D23-I236A/I237A/V239A (“236/237/239”), or TBC1D23-E425K/Y426A (“425/426”). The cells were fixed and labeled with anti-TGN46 (green) and GM130 (white) antibodies. Scale bar: 10 μm. (G) Quantitation of Golgi-localized TGN46 over its total amount in cells treated as in (F). Each dot represents results from one cell. *P* values were calculated using one-way ANOVA, post hoc Tukey’s test. ***P < 0.001. Experiments were triplicated, and the numerical data are included in S1 Data. aa, amino acid; FL, full-length; GAPDH, glyceraldehyde 3-phosphate dehydrogenase; GST, glutathione S-transferase; HEK, human embryonic kidney; KO, knockout; TBC, Tre2-Bub2-Cdc16; TGN, trans-Golgi network; ZFPL1, zinc finger protein like 1.

The results above suggested that TBC1D23 residues in both the TBC and rhodanese domains are involved in contacting with golgin-97/245 in vitro. To test whether these residues are relevant in vivo, we transfected various GST-tagged TBC1D23 constructs in HK293T cells and performed GST pull-down assays. As expected, TBC1D23 can effectively pulled down both endogenous or transfected golgin-97 (Fig 5A and S7 Fig). Deletion of the TBC domain led to a complete loss of binding to golgin-97, consistent with previous results that the TBC domain is necessary for the interaction between TBC1D23 and golgin-97 (Fig 5A and S7 Fig) [17]. Interestingly, deletion of the rhodanese domain also diminished the binding to golgin-97, suggesting that the rhodanese domain is also needed for the optimal interaction with golgin-97 or golgin-245 (Fig 5A and S7 Fig). Next, we introduced mutations in the context of TBC1D23 FL and assessed their golglin-97 binding ability. In line with binding experiments using purified proteins, mutations within either the TBC domain or the rhodanese domain significantly impaired the interaction between TBC1D23 and golgin-97 (Figs 4C, 4D and 5B). Finally, depletion of Arl1 led to decreased localization of TBC1D23 at the Golgi, consistent with the notion that Arl1 dictates the Golgi-location of golgin-97 and golgin-245, which in turn recruits TBC1D23 (S8 Fig) [17,18,26].

Previous studies suggest that TBC1D23 is localized at the TGN via binding to the N-terminus of golgin-97/245, and additional mechanisms, such as binding to PtdIns(4)P, are likely involved in TBC1D23 membrane targeting [17,18,33]. To assess the relevance of the binding to golgin-97/245 for the TGN accumulation of TBC1D23, we examined the subcellular localization of TBC1D23 FL and several mutants that are defective in binding to golgin-97. All mutations, including a mutation in the rhodanese domain (425/426), strongly reduced the colocalization with ZFPL1, relative to FL TBC1D23, revealed by quantitative image analysis (Fig 5C and 5D). To determine how the binding to golgin-97/245 impacts the cellular functions of TBC1D23, we chose TGN46 and CI-MPR as model cargoes. TBC1D23 is known to be required for their transport from endosome to TGN, and deletion of TBC1D23 led to their lysosomal degradation [17]. The reduction of TGN46 levels in the TBC1D23 knockout (KO) cells can be rescued by lentiviral reexpression of mCherry-TBC1D23 WT but not by the mutants that are defective in binding to golgin-97/245 (Fig 5E). This assay is further complemented by quantitative immunofluorescence analysis of TGN46 on the Golgi, which revealed that TBC1D23 WT, but not these mutants, could effectively restore the accumulation of TGN46 on the Golgi (Fig 5F and 5G). Similarly, disruption of the binding to golgin-97/245 perturbed the endosome-to-TGN trafficking of another well-established cargo, CI-MPR, and led to its lysosomal degradation (S9 Fig). Taken together, our data demonstrate that golgin-97/245 binds to both the TBC and rhodanese domains of TBC1D23 and that this binding is critical for both the subcellular localization and cellular functions of TBC1D23.

### Binding to golgin-97/245 is required for motor neuronal development in zebrafish

**Fig 6:**
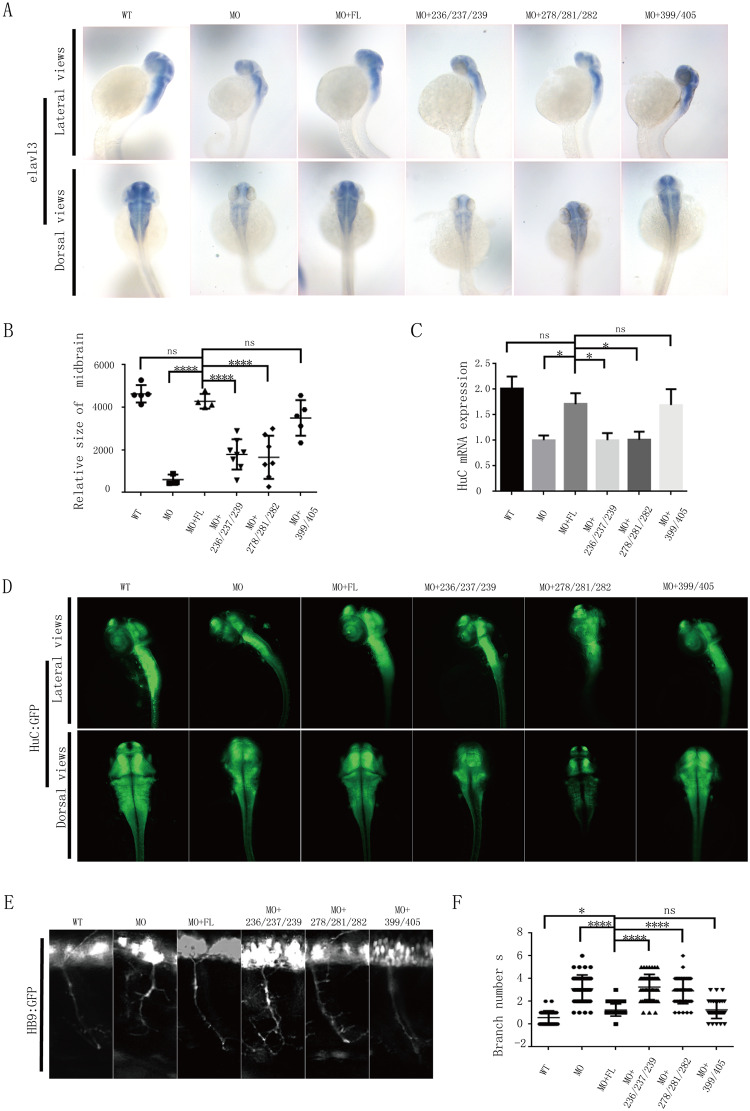
Disruption of TBC1D23-golgin-97/245 interaction impairs neuronal growth and brain development in zebrafish. (A) HuC (elavl3) spatial expression in zebrafish at 48 hpf, determined by in situ hybridization. MO: MO injection; MO+ FL: MO and human FL TBC1D23 mRNA co-injection; MO+278/281/282: MO and human TBC1D23 L278A/Y281A/Y282A mutant mRNA co-injection; MO+236/237/239: MO and human TBC1D23 I236A/I237A/V239A mutant mRNA co-injection; MO+399/405: MO and human TBC1D23 C399S/R405A mutant mRNA co-injection. All injections are performed at the one-cell stage Top, lateral view; bottom, dorsal view. (B) The relative size of zebrafish midbrain. The size of the midbrain was measured from lateral view, and 5 to 10 embryos from each group were used for comparison. Data are presented as mean ± S.E.M. ****P < 0.0001. *P* values were calculated using one-way ANOVA, Tukey’s multiple-comparisons test. Experiments were triplicated, and the numerical data are included in S1 Data. (C) Relative transcription level of HuC at 48 hpf by semiquantitative RT-PCR analysis. Mean ± S.E.M. *P < 0.05. *P* values were calculated using one-way ANOVA, Tukey’s multiple-comparisons test. Experiments were triplicated, and the numerical data are included in S1 Data. (D) HuC (green) expression in Tg[HuC: GFP] transgenic zebrafish at 48 hpf, determined by immunofluorescence. Top, lateral view; bottom, dorsal view. (E) Morphology of CaP axons from embryos at 48 hpf that were injected MO and/or different mRNA. All injections are performed at the one-cell stage of the Tg[hb9: GFP]^ml2^ transgenic zebrafish embryos. Arrows indicate abnormal branches. Lateral views and enlarged views are shown. (F) Statistical results of the branch number of CaP axons in embryos treated as in (E). For each group, approximately 45 axons from nine Tg[hb9: GFP]^ml2^ transgenic zebrafish embryos are scored. Mean ± S.E.M. ****P < 0.0001,*P < 0.05. *P* values were calculated using one-way ANOVA, Tukey’s multiple-comparisons test. Experiments were triplicated, and the numerical data are included in S1 Data. FL, full-length; hpf, hours post fertilization; MO, morpholino oligonucleotide; ns, not significant; RT-PCR, reverse transcription PCR; WT, wild type.

Homozygous mutations of TBC1D23 have been recently linked with PCH [18,20]. We and others have shown that knockdown of TBC1D23 in zebrafish can mimic many features of the PCH patients, including reduced brain size, altered brain structure, impaired mobility, and abnormal neuronal development [18,33]. Thus, we chose zebrafish as a model organism and tested the requirement of golgin-97/245 binding for zebrafish neuronal development. Depletion of TBC1D23 by morpholino oligonucleotide (MO) injection led to a reduction of HuC mRNA expression, a marker of early neurons that are critical for neuronal development (Fig 6A) [33,44]. The reduction can be rescued by co-injection of FL TBC1D23 mRNA; in contrast, both mutants defective in golgin-97/245-binding failed to increase HuC expression (Fig 6A). Similarly, the reduction of midbrain size caused by MO injection could be rescued by FL TBC1D23 but not by either 278/281/282 or 236/237/239 triple mutants (Fig 6A and 6B). These observations were further corroborated by semiquantitative reverse transcription PCR (RT-PCR) and whole-mount immunofluorescence analysis, which revealed a similar trend (Fig 6C and 6D). In contrast with these triple mutants, the 399/405 mutant could restore HuC mRNA expression, protein expression, and midbrain size, similar to FL TBC1D23 (Fig 6A–6D). These results are consistent with the notion that the rhodanese domain is unlikely to be enzymatically active.

We have previously reported that TBC1D23 is critical for the development of CaP motor neuron axons during embryonic neurogenesis [33]. To probe whether the TBC1D23-golgin-97/245 binding is important for zebrafish neuronal development, we tested the effect of the 278/281/282 and 236/237/239 mutants on the CaP axon morphology. Knockdown of TBC1D23 led to abnormal CaP axon morphology and increasing branching number, with the average branching numbers per embryo in WT and MO being 0.56 and 3.1, respectively (Fig 6E and 6F). Embryos co-injected with MO and the FL mRNA restored axon morphology and branching number (Fig 6E and 6F). Conversely, co-injection of either the 278/281/282 or 236/237/239 mutants resulted in a defective morphology, similar to that of MO alone (Fig 6E and 6F). Consistent with our biochemical and cellular studies, the 399/405 mutant displayed a normal CaP axon morphology (Fig 6E and 6F). Taken together, our data indicated that the TBC1D23-golgin-97/245-binding is required for motor neuronal development in zebrafish and that the putative catalytic site of the rhodanese domain is dispensable in the process.

## Discussion

The Rab family of small GTPases plays a central role in vesicle-mediated membrane trafficking, and members of the TBC family often function in membrane trafficking through regulating the activation states of Rabs. Unlike many other members of the TBC family, TBC1D23 is unable to promote GTP hydrolysis by Rabs [18]. However, TBC1D23 facilitates endosome-to-Golgi trafficking through interacting with Golgi-bound golgin-97/245 and the WASH/FAM21 complex localized on endosomal vesicles [17,19]. Furthermore, homozygous mutations in TBC1D23 lead to the human developmental disorder PCH [18,20]. To gain further mechanistic insights into the roles of TBC1D23 in trafficking and development, we present the crystal structure of the TBC and rhodanese domains of TBC1D23, discover that the rhodanese domain of TBC1D23 unlikely functions as an active phosphatase or sulfurtransferase, and define the binding sites of golgin-97/245 on TBC1D23. Selective disruption of the golgin-97/245-binding site results in defects in both endosomal trafficking in cells and neuronal development in zebrafish.

The rhodanese domain is an ancient protein fold that can be found in all three kingdoms. Based on the presence of other domains, the rhodanese-containing proteins can be classified as (1) single domain, (2) tandem domains, or (3) combined with other protein domain [30,31]. Biochemically, rhodanese domains can be classified as enzymatically active and associate with activities such as protein phosphatase or sulfurtransferase, or as enzymatically inactive. In most cases, the active and inactive rhodanese domains can be easily distinguished by their aa sequences, as the active rhodanese domain possesses a characteristic catalytic cysteine residue [30,31]. Our structural and biochemical analysis revealed that TBC1D23 unlikely functions as an active phosphatase or sulfurtransferase, despite containing a putative active-site cysteine. Instead, the rhodanese domain functions with the TBC domain, and the two domains unitedly form the platform to accommodate the N-terminus of golgin-97/245. Our biochemical, cellular, and zebrafish studies collectively demonstrated that impacting the golgin-97/245 binding led to defective endosomal trafficking and abnormal brain development.

Together with previous studies [17,26–28,33], our work provides a model on the recruitment and functions of TBC1D23: (1) activated Arl1 GTPase recruits golgin-97/245 by interacting with their C-terminal GRIP domain; (2) the N-terminus of golgin-97/245 is extended a few hundred nanometers from the TGN, because of the presence of their coiled-coil regions, and interacts with the TBC and rhodanese domains of TBC1D23; (3) the C-terminal PH-like domain of TBC1D23 promotes vesicle recruitment by associating with FAM21 and PtdIns(4)P. Whereas our current study provided some new insights into functions of TBC1D23 in trafficking and development, we are left with many more important questions. First, how do different regions of TBC1D23 and golgin-97/245 function together to bring vesicles in proximity to the membrane? Second, are there other enzymatically inactive TBC-containing proteins that function in membrane trafficking, similar to TBC1D23? Last but not least, what are the mechanisms by which defects in membrane trafficking between endosomes and TGN result in defects in neuronal development? Answering these questions will be of interest for the fields of membrane trafficking and neurological diseases, and our current molecular understanding of TBC1D23 lays a foundation for these exciting future discoveries.

## Methods

### Antibodies and plasmids

DNA constructs and antibodies used in this paper were listed in S2 and S3 Tables, respectively.

### Cloning, expression, and purification

The DNA sequence encoding human TBC1D23 aa1–460 (D23^N^) was cloned into a modified pGEX-4T1 vector to express a protein with an N-terminal cleavable GST fusion. Protein was expressed in *Escherichia coli* BL21 (DE3) strain at 20 °C for 12 h. The protein was first captured by glutathione-Sepharose beads. After extensive washes, the bead-bound protein was subjected to on-column TEV cleavage. The eluate was collected, concentrated, and further purified by size exclusion chromatography using a Superdex 200 column (GE Healthcare) in 20 mM Tris (pH 8.0), 200 mM NaCl, and 5 mM DTT. The seleno-methionine (SeMet) substituted D23^N^ was purified using an identical protocol, which yielded about one-third of the native protein.

TSTD1 was cloned into a pET-28b vector downstream of a hexahistidine tag. Protein was expressed at 16 °C for 18 h. The protein was purified by Ni-NTA beads and eluted with a Ni-B buffer (50 mM Tris [pH 8.0], 150 mM NaCl, 300 mM imidazole). The eluate was collected and dialyzed against a buffer without 300 mM imidazole.

CDC25B was cloned into a modified pGEX-4T1 vector, yielding an N-terminal cleavable GST fusion. The protein was purified similar to D23^N^, and the GST fusion was removed for the enzymatic assay.

### Crystallization and data collection

Crystals of both the native and the SeMet-derivative proteins of D23^N^ were grown using the hanging-drop vapor-diffusion methods at 18 °C, by mixing equal amounts of D23^N^ protein solution (12 mg/mL) and reservoir buffer containing 0.1 M Tris (pH 8.5), 0.4 M magnesium chloride, 25% (w/v) polyethylene glycol 3350. All crystals were cryo-protected using reservoir buffer supplemented with 20% (v/v) glycerol and flash-frozen in liquid nitrogen. X-ray diffraction data was collected at Shanghai Synchrotron Radiation facility beamline BL17U1. The data collection statistics are given in S1 Table.

### Structure solution and refinement

Structure was solved by Se-SAD. The resulting phase was used to allow modeling of one D23^N^ molecule through the program Autobuild [45] and manual building using the program COOT [46]. Refinement was performed using the program Refmac5 [47]. Coordinates were deposited with accession codes 6JL7.

### ITC

ITC experiments were performed on a Microcal iTC200 instrument, as previously described [12,48]. All the proteins and peptides used for ITC experiments were dialyzed against the ITC buffer (20 mM Tris [pH 8.0], 200 mM NaCl), prior to the titration. Golgin-97 and golgin-245 peptides (0.5 mM) were titrated into D23^N^ protein (50 μM) at 25 °C. Each experiment was performed at least two times.

### Pull-down experiments

GST pull-down experiments were carried out as previously described [48,49]. The mixture contained 20 μg of GST or GST-tagged protein and 400 μg of bait proteins. The proteins were mixed with glutathione-Sepharose beads (30 μl) in 0.5 ml of pull-down buffer (20 mM Tris [pH 8.0], 200 mM NaCl, 5mM DTT, 0.005% Triton-X100) and allowed to bind at 4 °C for 1 h. The beads were then washed three times with 1 mL of pull-down buffer. The bound samples were separated on 15% SDS-PAGE and visualized by Coomassie staining.

For pull-down assays using cell lysate, HEK 293T cells (ATCC) were transfected with plasmids encoding GST or GST-tagged golgin-97. Whole-cell lysate was prepared by adding lysis buffer (50 mM Tris [pH 8.0], 5 mM EDTA, 150 mM NaCl, 0.5% [v/v] NP-40) and 1% protein inhibitor (Bimake, B14002). Cells were lysed by ultrasonication and then centrifuged at 10,000 rpm for 5 min. Supernatant was incubated with glutathione-Sepharose beads at 4 °C for 3 h, and beads were washed with 1 mL of lysis buffer four times. Bead-bound samples were detected by immunoblotting using the indicated antibodies (summarized in S3 Table).

### GAP activity assay

The GAP assay was carried out using the EnzChek Phosphate Assay Kit (Invitrogen), similar to previous studies [12,50]. Briefly, RAB proteins were purified and incubated with 15-fold molar excess of GTP at room temperature for 30 min. After incubation, free nucleotides were removed by passing a desalting column. GTP-preloaded Rabs and purified TBC domain of TBC1D23 were prepared in two different solutions. The reaction was initiated by mixing the two solutions, and the final solution contained 20 mM HEPES (pH 7.5), 150 mM NaCl, 10 mM MgCl2, 200 mM 2-amino-6-mercapto-7-methylpurine riboside (MESG), 5 units of purine nucleoside phosphorylase, 20 mM Rab, and various amounts of TBC1D23. The absorbance at 360 nm was continuously monitored, and kinetics was determined similarly to previous studies [50].

### Sulfurtransferase assays

The sulfurtransferase assays were performed as described previously [42]. The first assay used thiosulfate as sulfur donor, which was converted into H_2_S under the catalysis of sulfurtransferase. H_2_S was further converted into lead sulfide, which has absorption at 390 nm. The reaction mixture contained GSH (50 mM), thiosulfate (20 mM), and lead acetate (0.4 mM) in 100 mM HEPES (pH 7.5), 150 mM NaCl (250 μl final volume). The reaction was initiated by the addition of enzyme (10 μg). After incubation at 37 °C for 5 min, absorbance at 390 nm was recorded.

The second assay used contained thiosulfate and potassium cyanide as substrates. The reaction mixture contained thiosulfate (20 mM), potassium cyanide (20 mM), and purified TSTD1 protein (10 μg) in 100 mM HEPES (pH 7.5), 150 mM NaCl, 100 mM HEPES (pH 7.5). After reaction at 37 °C for 20 min, the reaction was quenched by the addition of formaldehyde, followed by a ferric nitrate solution, as descripted in previous studies [42]. Absorbance at 460 nm was recorded.

### Phosphatase assay

pNPP was utilized as a substrate in the phosphatase assay, similar to previous studies [41,51]. The reaction mixture contained 100 μM pNPP and 25 μg of protein in a buffer containing 50 mM Tris (pH 9.0), 50 mM NaCl, 0.005% Tween, 1 mM EDTA, and 1 mM DTT. Reaction was carried at 25 °C for 30 min, and absorbance at 405 nm was measured.

### Cell culture, immunofluorescent staining, and confocal microscopy

HEK 293T and HeLa cell lines were obtained from ATCC and cultured in DMEM supplemented with 10% fetal bovine serum (BI) at 37 °C. The PEI transfection system (Invitrogen) was used for transfection. TBC1D23 KO cell lines were generated similar to the previous study, using the target guide RNA 5′-CTGCCAACGTCGAGCGGCGA-3′ [17,33].

Immunofluorescence experiments were performed as previously described [36,52]. Samples were fixed by 4% formaldehyde in PBS, followed by permeabilization with 0.15% Surfact-Amps X-100 (Thermo Fisher) in PBS. Samples were then incubated overnight at 4 °C with primary antibodies. After washing, the samples were incubated with secondary antibodies for 1 h at room temperature or overnight at 4 °C. Antibodies used in our study are summarized in the S3 Table. Confocal images were acquired by Zeiss LSM 780 and Olympus FV-1000 confocal microscope and were analyzed using NIH ImageJ software. All microscopic experiments were conducted at least three times.

### Zebrafish

All zebrafish (*Danio rerio*) experiments were performed in accordance with the guidelines of the animal ethical committee of West China Hospital [33, 53]. In this study, AB strain (WT), Tg[HuC:GFP] strain (transgenic), and Tg[Hb9:GFP] strains were used [44,54].

### Morpholino and mRNA injections

Two different of antisense MOs were used as previously described [18,33]. Although injection of the two MOs led to similar phenotypes, the translation-blocking MO was used for most of the current study, as it is more efficient to inhibit TBC1D23 [33]. At the one- to two-cell stage of development, MO (7.5 ng) and/or mRNA (150 ng) were injected into yolk or cells.

### Total RNA isolation and semiquantitative RT-PCR

Total RNA of about 40 zebrafish embryos at 48 hpf was isolated as previously described [33,53]. Briefly, the embryos were grounded, and tissue debris was removed by centrifugation (13,000 rpm for 1 min). Total RNA was isolated using RNeasy Plant Mini Kit (FOREGRNE). RNA samples at the same concentrations were used to synthesize cDNA using the Prime Script Reverse-Transcription PCR kit (TaKaRa DRR014A). CFX96 real-time system (BIORAD) was used for the PCR reaction using the resultant cDNA as template (200 ng per sample). Sequences of primers are shown in S4 Table.

### Whole-mount in situ hybridization (WISH)

WISH experiments were performed as previously described [33,53]. In brief, zebrafish embryos at 48 hpf were permeabilized with Proteinase K (10 mg/ml, Promega) for 30 min. Samples were then incubated with the digoxygenin (DIG)-labeled antisense probes at 65 °C overnight. Probes were then removed, and the embryos were incubated with alkaline phosphatase (AP)-conjugated anti-DIG antibody (1:3,000) at 4 °C overnight. After washing, the samples were subjected to NBT/BCIP (Roche) staining according to the manufacturer’s instructions. The antisense RNA probe (elavl3/HuC) was synthesized using a DIG RNA labeling kit (Roche).

## Supporting information

S1 Fig Interaction between the TBC and rhodanese domains of TBC1D23 and structural comparsion of the TBC domains of TBC1D23 and Gyp1p. (A) Detailed view of the interactions between the TBC and rhodanese domains of TBC1D23. The TBC domain is shown in electrostatic potential surface representation (Blue: positive potential; red: negative potential), and rhodanese domain (yellow) is shown in ribbon representation. The complex is shown in the same orientation as that of the left molecule in Fig 1A. Selective residues forming intramolecular hydrogen bonds (dashed line) and van der Waals interactions are shown on the left and right of the main figure, respectively. Residues from the TBC and rhodanese domain are colored in cyan and gold and labeled with blue and black fonts, respectively. (B) Overlay of the structure of the TBC domain of TBC1D23 with the Gyp1p-Rab33 complex by superimposing the TBC domain. Green: Rab33; cyan: TBC domain of TBC1D23; gray: TBC domain of Gyp1p. (C) Comparison of the active site of Gyp1p and the corresponding residues of TBC1D23, with residues from Gyp1p and TBC1D23 colored in gray and cyan and labeled with black and blue fonts, respectively. TBC, Tre2-Bub2-Cdc16.
(TIF)

S2 Fig Sequence comparison of the N-terminus of TBC1D23 from different model organisms. Sequence alignments were performed with ClustalW, with protein secondary structure listed above and consensus sequence listed below. ●, putative catalytic residues of the rhodanese domain; ▲, golgin-97/245-binding.
(TIF)

S3 Fig GAP activity of the TBC domains of TBC1D23 and TBC1D5. Catalytic efficiency (*k*_cat_/K_M_) relative to the intrinsic rate constant (*k*_intr_) for GTP hydrolysis was calculated from two replicate experiments (mean ± S.D.), and the numerical data are included in S1 Data. GAP, GTPase-activating protein; TBC, Tre2-Bub2-Cdc16.
(TIF)

S4 Fig The thiosulfate:cyanide sulfurtransferase activity of TSTD1 WT, C79S, and TBC1D23 (aa1–460). Absorbance at 460 nm was monitored. Data are from three replicate experiments (mean ± S.D.), and the numerical data are included in S1 Data. aa, amino acid; TSTD1, thiosulfate sulfurtransferase like domain containing 1; WT, wild type.
(TIF)

S5 Fig Helical wheel representation of the N-terminal 14 residues of golgin-97/245, with the numbers inside the circle indicating the residue sequence in the protein. Three residues critical for contacting TBC1D23 are indicated: F in position 2, L in position 5, and I or L in position 9.
(JPG)

S6 Fig In vitro interactions between TBC1D23 and golgin-97/245. (A) aa sequences of golgin-97 peptides used for ITC experiments in (B) and for the GST fusion (C). (B) GST pull-down assays performed with GST-golgin-97-25aa, -12aa, -8aa, or GST, and purified D23^N^. After incubation with soluble protein(s), the resin was extensively washed. The purified proteins used in the assays (left) and resin-bound proteins (right) were then subjected to SDS-PAGE and Coomassie Blue staining. (C) ITC experiments for the binding of the golgin-97-25aa, -12aa, or -8aa peptide with D23^N^. Top and bottom panels show raw and integrated heat from injections, respectively. The solid curves in the bottom panel represent a fit of the integrated data to a single-site binding model. (D) GST pull-down assays performed with GST-golgin-245-25aa, and purified D23^N^ WT, E179K (“179”), L278A/Y281A/Y282A (“278/281/282”), I236A/I237A/V239A (“236/237/239”), E396K (“396”), or TBC1D23-E425K/Y426A (“425/426”). After incubation with soluble protein(s), the resin was extensively washed. The resin-bound proteins were then subjected to SDS-PAGE and Coomassie Blue staining. (E) GST pull-down assays performed with GST-golgin-97-25aa, 25aa-K6E/K8E, or GST, and purified D23^N^. After incubation with soluble protein(s), the resin was extensively washed. The resin-bound proteins were then subjected to SDS-PAGE and Coomassie Blue staining. GST pull-down assays performed with GST-golgin-97-25aa, or GST, and purified human, fly, and worm D23^N^ WT or mutants (fly MUT: I223A/I224A/I226A; worm MUT: V220A/F221A/V223A). After incubation with soluble protein(s), the resin was extensively washed. The purified proteins used in the assays (left) and resin-bound proteins (right) were then subjected to SDS-PAGE and Coomassie Blue staining. D23^N^, N-terminus of TBC1D23; GST, glutathione S-transferase; ITC, isothermal titration calorimetry.
(TIF)

S7 Fig Interactions between TBC1D23 and golgin-97 in cells. GST pull-down analyses of HEK 293T cells transfected with vectors expressing GST, GST-TBC1D23-FL (“FL”), GST-TBC1D23-ΔTBC, GST-TBC1D23-ΔRhod1, or GST-TBC1D23-TBC+Rhod1, and Myc-tagged golgin-97. Cell lysates were precipitated with glutathione-Sepharose beads and probed with anti-GST, Myc (to detect golgin-97), or GAPDH (control) antibodies. Top: input samples; bottom: bound samples. FL, full-length; GAPDH, glyceraldehyde 3-phosphate dehydrogenase; GST, glutathione S-transferase; HEK, human embryonic kidney.
(TIF)

S8 Fig Arl1 controls the Golgi localization of TBC1D23. (A) Confocal immunofluorescence of HeLa cells transfected with siRNA targeting Arl1 or control siRNA. Cells were fixed 72 h after transfection and labeled with anti-CI-MPR (green) and GM130 (white) antibodies. Scale bar: 10 μm. (B) Quantitation of TBC1D23 colocalization with GM130 in cells as treated in (A); each point represents one cell. *P* values were calculated using unpaired *t* test. ***P < 0.0001. Experiments were triplicated, and the numerical data are included in S1 Data. (C) Immunoblot of whole-cell extracts for cells as treated in (A), showing the total protein levels of Arl1, golgin-97, and TBC1D23. CI-MPR, cation-independent mannose-6-phosphate receptor; siRNA, small interfering RNA.
(TIF)

S9 Fig Interaction between TBC1D23 and golgin-97/245 is required for CI-MPR retrograde trafficking. (A) Confocal immunofluorescence of TBC1D23 knockout HeLa cells transfected with vectors expressing mCherry, mCherry-TBC1D23-FL (“FL”), TBC1D23-L278A/Y281A/Y282A (“278/281/282”), TBC1D23-I236A/I237A/V239A (“236/237/239”), or TBC1D23-E425K/Y426A (“425/426”). The cells were fixed and labeled with anti-CI-MPR (green) and ZFPL1 (white) antibodies. Scale bar: 10 μm. (B) Quantitation of Golgi-localized CI-MPR over its total amount in cells treated as in (A). Each dot represents result from one cell. *P* values were calculated using one-way ANOVA, post hoc Tukey’s test. ***P < 0.0001. Experiments were triplicated, and the numerical data are included in S1 Data. (C) Immunoblot of whole-cell extracts of TBC1D23 knockout HeLa cells transfected with vectors expressing mCherry, mCherry-TBC1D23-FL (“FL”), TBC1D23-L278A/Y281A/Y282A (“278/281/282”), TBC1D23-I236A/I237A/V239A (“236/237/239”), or TBC1D23-E425K/Y426A (“425/426”). Cell lysates were probed with anti-cherry, TBC1D23, CI-MPR, or tubulin (control) antibodies. CI-MPR, cation-independent mannose-6-phosphate receptor; FL, full-length; ns, not significant; siRNA, small interfering RNA; ZFPL1, zinc finger protein like 1.
(TIF)

S1 Table Crystallography data collection and refinement statistics. (DOCX)

S2 Table DNA constructs used in this study. (DOCX)

S3 Table Summary of antibodies used in this study. (DOCX)

S4 Table Sequences of primers, morpholino, and siRNA. siRNA, short interfering RNA.
(DOCX)

S1 Raw images Unprocessed images of all gels and blots in the paper. (PDF)

S1 Data Numerical data for Figs 2C, 2D, 3B, 3D, 4D, 5D, 5G, 6B, 6C and 6F, S3, S4, S6C, S8B and S9B Figs. (XLSX)

## Abbreviations

aa: amino acid
CI-MPR: cation-independent mannose-6-phosphate receptor
D23^N^: N-terminus of TBC1D23
FL: full-length
GAPDH: glyceraldehyde 3-phosophate dehydrogenase
GST: glutathione S-transferase
GTP: GTPase-activating protein
ITC: isothermal titration calorimetry
KO: knockout
MO: morpholino oligonucleotide
mTOR: mammalian target of rapamycin
PCH: pontocerebellar hypoplasia
PDB: Protein Data Bank
PH: Pleckstrin homology
pNPP: disodium 4-nitrophenyl phosphate
PtdIns(4)P: phosphatidylinositol 4-phosphate
rmsd: root-mean-square deviation
RT-PCR: reverse transcription PCR
Se-SAD: selenium single-wavelength anomalous diffraction
TBC: Tre2-Bub2-Cdc16
TGN: trans-Golgi network
TSTD1: thiosulfate sulfurtransferase like domain containing 1
WT: wild type
ZFPL1: zinc finger protein like 1
