# Supplementary figures and images for: Structure of TBC1D23 N-terminus reveals a novel role for rhodanese domain
Source: PLoS Biol. 2020 May 26;18(5):e3000746. doi: 10.1371/journal.pbio.3000746 (PMC7274447; doi:10.1371/journal.pbio.3000746)

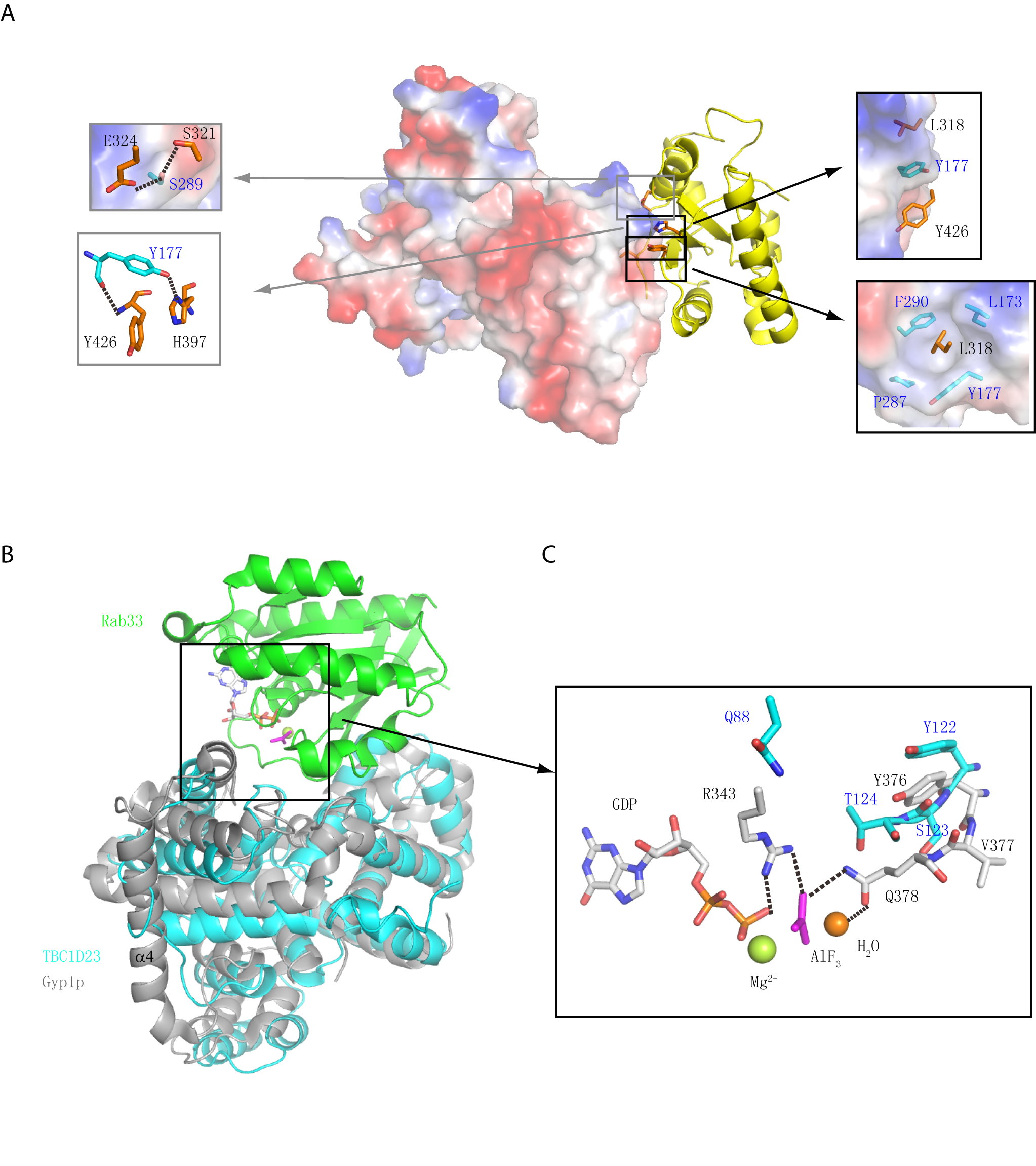

Supplement: S1 Fig — (A) Detailed view of the interactions between the TBC and rhodanese domains of TBC1D23. The TBC domain is shown in electrostatic potential surface representation (Blue: positive potential; red: negative potential), and rhodanese domain (yellow) is shown in ribbon representation. The complex is shown in the same orientation as that of the left molecule in Fig 1A. Selective residues forming intramolecular hydrogen bonds (dashed line) and van der Waals interactions are shown on the left and right of the main figure, respectively. Residues from the TBC and rhodanese domain are colored in cyan and gold and labeled with blue and black fonts, respectively. (B) Overlay of the structure of the TBC domain of TBC1D23 with the Gyp1p-Rab33 complex by superimposing the TBC domain. Green: Rab33; cyan: TBC domain of TBC1D23; gray: TBC domain of Gyp1p. (C) Comparison of the active site of Gyp1p and the corresponding residues of TBC1D23, with residues from Gyp1p and TBC1D23 colored in gray and cyan and labeled with black and blue fonts, respectively. TBC, Tre2-Bub2-Cdc16. (TIF) [file pbio.3000746.s001.tif]

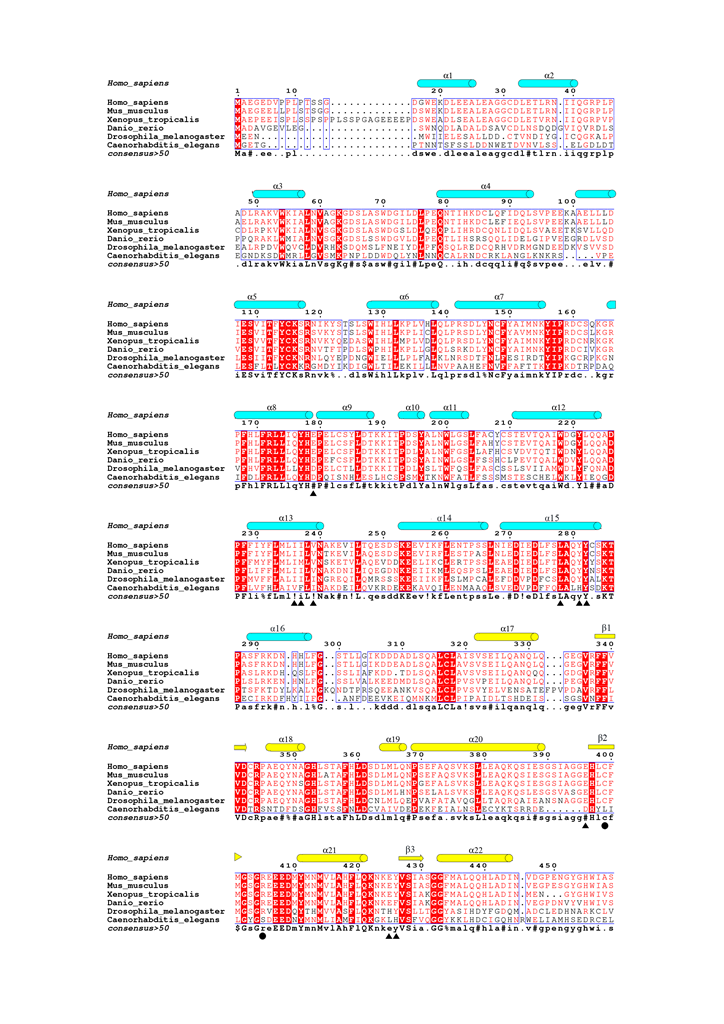

Supplement: S2 Fig — Sequence alignments were performed with ClustalW, with protein secondary structure listed above and consensus sequence listed below. ●, putative catalytic residues of the rhodanese domain; ▲, golgin-97/245-binding. (TIF) [file pbio.3000746.s002.tif]

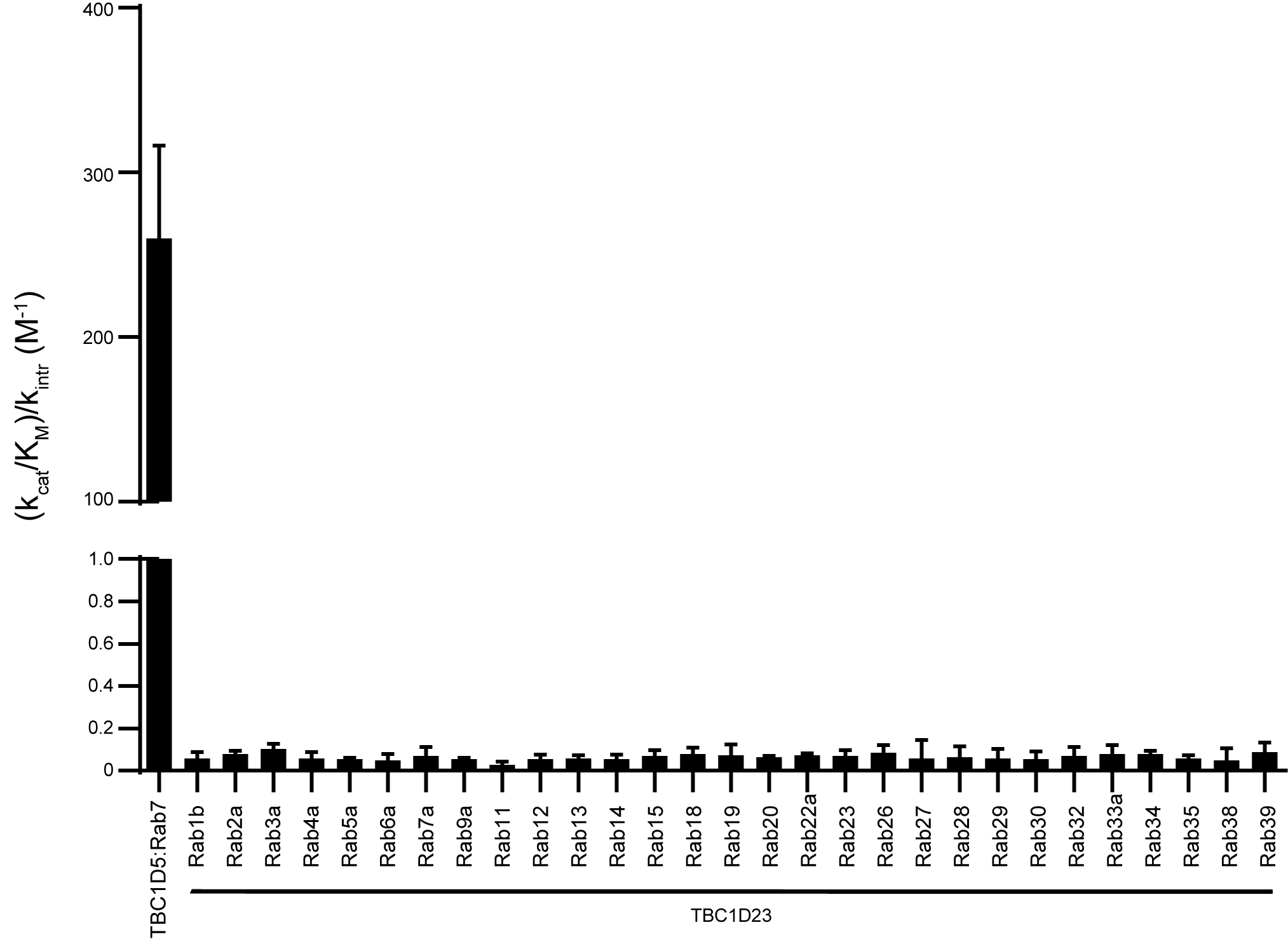

Supplement: S3 Fig — Catalytic efficiency (kcat/KM) relative to the intrinsic rate constant (kintr) for GTP hydrolysis was calculated from two replicate experiments (mean ± S.D.), and the numerical data are included in S1 Data. GAP, GTPase-activating protein; TBC, Tre2-Bub2-Cdc16. (TIF) [file pbio.3000746.s003.tif]

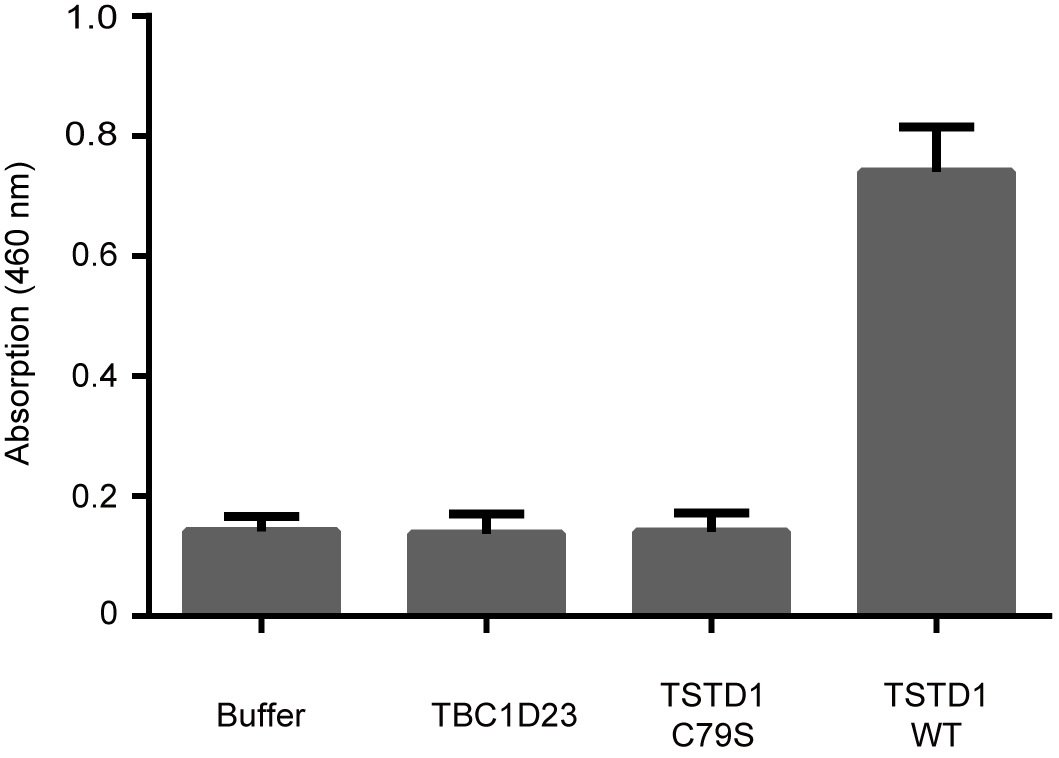

Supplement: S4 Fig — Absorbance at 460 nm was monitored. Data are from three replicate experiments (mean ± S.D.), and the numerical data are included in S1 Data. aa, amino acid; TSTD1, thiosulfate sulfurtransferase like domain containing 1; WT, wild type. (TIF) [file pbio.3000746.s004.tif]

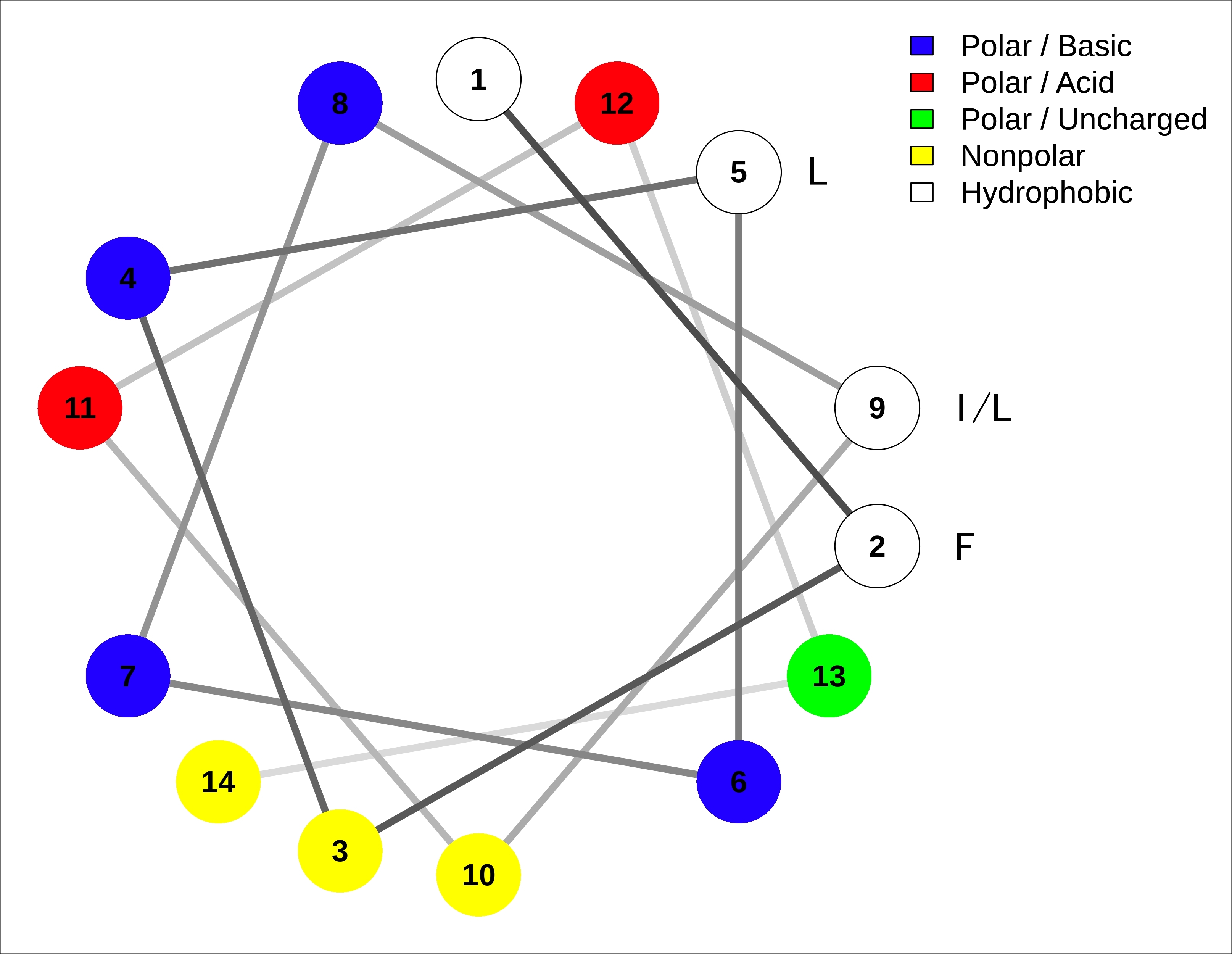

Supplement: S5 Fig — Three residues critical for contacting TBC1D23 are indicated: F in position 2, L in position 5, and I or L in position 9. (JPG) [file pbio.3000746.s005.jpg]

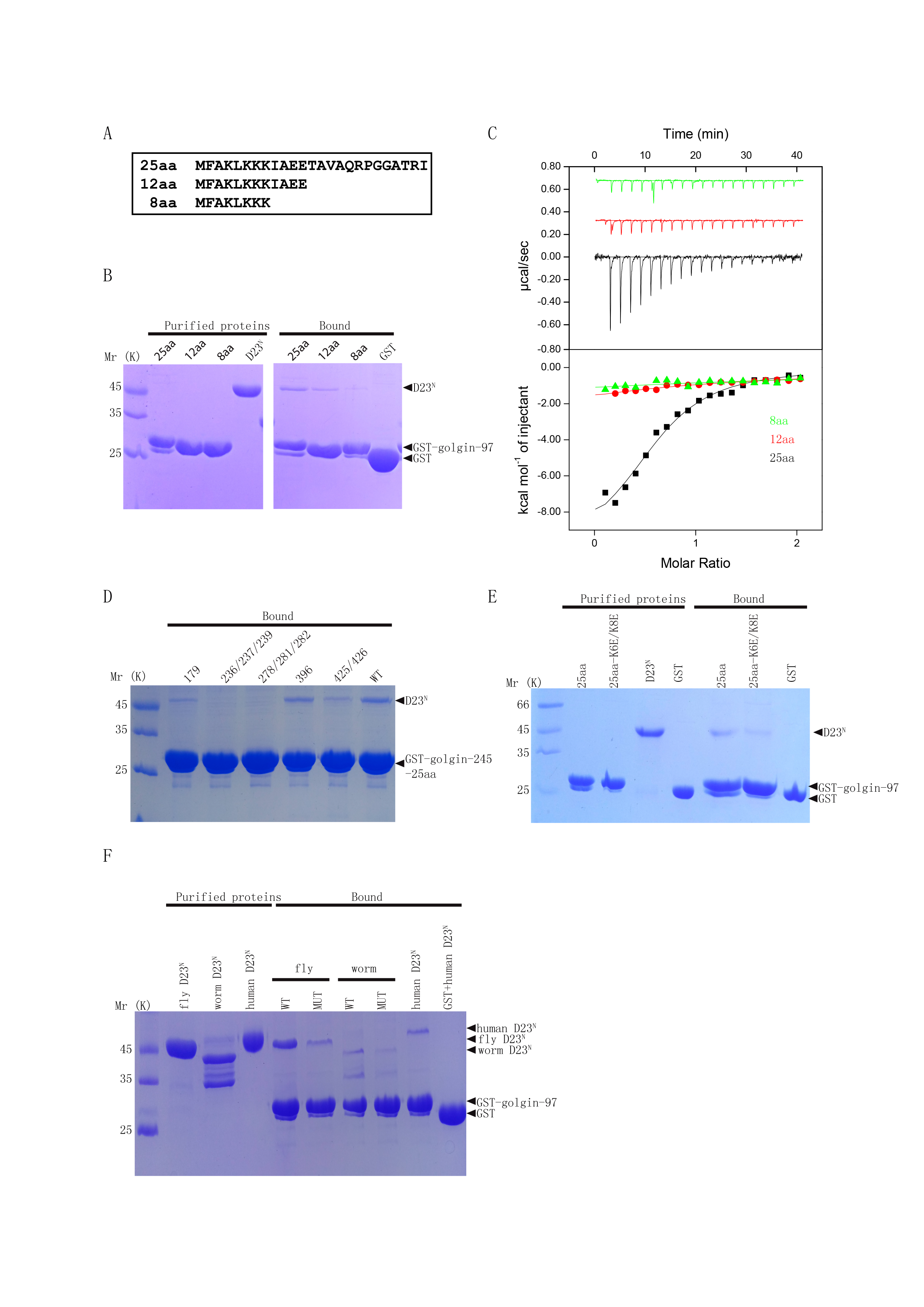

Supplement: S6 Fig — (A) aa sequences of golgin-97 peptides used for ITC experiments in (B) and for the GST fusion (C). (B) GST pull-down assays performed with GST-golgin-97-25aa, -12aa, -8aa, or GST, and purified D23N. After incubation with soluble protein(s), the resin was extensively washed. The purified proteins used in the assays (left) and resin-bound proteins (right) were then subjected to SDS-PAGE and Coomassie Blue staining. (C) ITC experiments for the binding of the golgin-97-25aa, -12aa, or -8aa peptide with D23N. Top and bottom panels show raw and integrated heat from injections, respectively. The solid curves in the bottom panel represent a fit of the integrated data to a single-site binding model. (D) GST pull-down assays performed with GST-golgin-245-25aa, and purified D23N WT, E179K (“179”), L278A/Y281A/Y282A (“278/281/282”), I236A/I237A/V239A (“236/237/239”), E396K (“396”), or TBC1D23-E425K/Y426A (“425/426”). After incubation with soluble protein(s), the resin was extensively washed. The resin-bound proteins were then subjected to SDS-PAGE and Coomassie Blue staining. (E) GST pull-down assays performed with GST-golgin-97-25aa, 25aa-K6E/K8E, or GST, and purified D23N. After incubation with soluble protein(s), the resin was extensively washed. The resin-bound proteins were then subjected to SDS-PAGE and Coomassie Blue staining. GST pull-down assays performed with GST-golgin-97-25aa, or GST, and purified human, fly, and worm D23N WT or mutants (fly MUT: I223A/I224A/I226A; worm MUT: V220A/F221A/V223A). After incubation with soluble protein(s), the resin was extensively washed. The purified proteins used in the assays (left) and resin-bound proteins (right) were then subjected to SDS-PAGE and Coomassie Blue staining. D23N, N-terminus of TBC1D23; GST, glutathione S-transferase; ITC, isothermal titration calorimetry. (TIF) [file pbio.3000746.s006.tif]

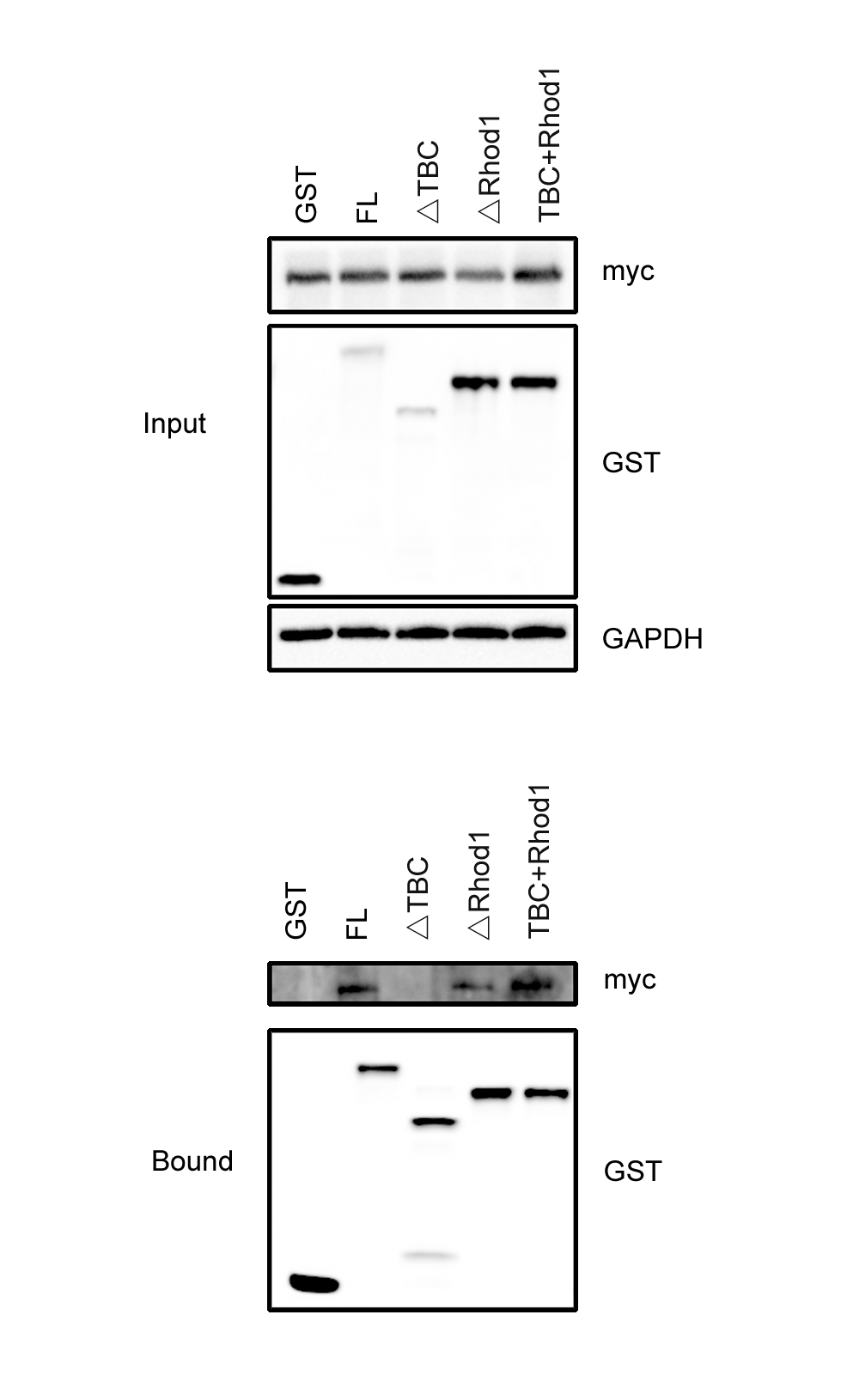

Supplement: S7 Fig — GST pull-down analyses of HEK 293T cells transfected with vectors expressing GST, GST-TBC1D23-FL (“FL”), GST-TBC1D23-ΔTBC, GST-TBC1D23-ΔRhod1, or GST-TBC1D23-TBC+Rhod1, and Myc-tagged golgin-97. Cell lysates were precipitated with glutathione-Sepharose beads and probed with anti-GST, Myc (to detect golgin-97), or GAPDH (control) antibodies. Top: input samples; bottom: bound samples. FL, full-length; GAPDH, glyceraldehyde 3-phosphate dehydrogenase; GST, glutathione S-transferase; HEK, human embryonic kidney. (TIF) [file pbio.3000746.s007.tif]

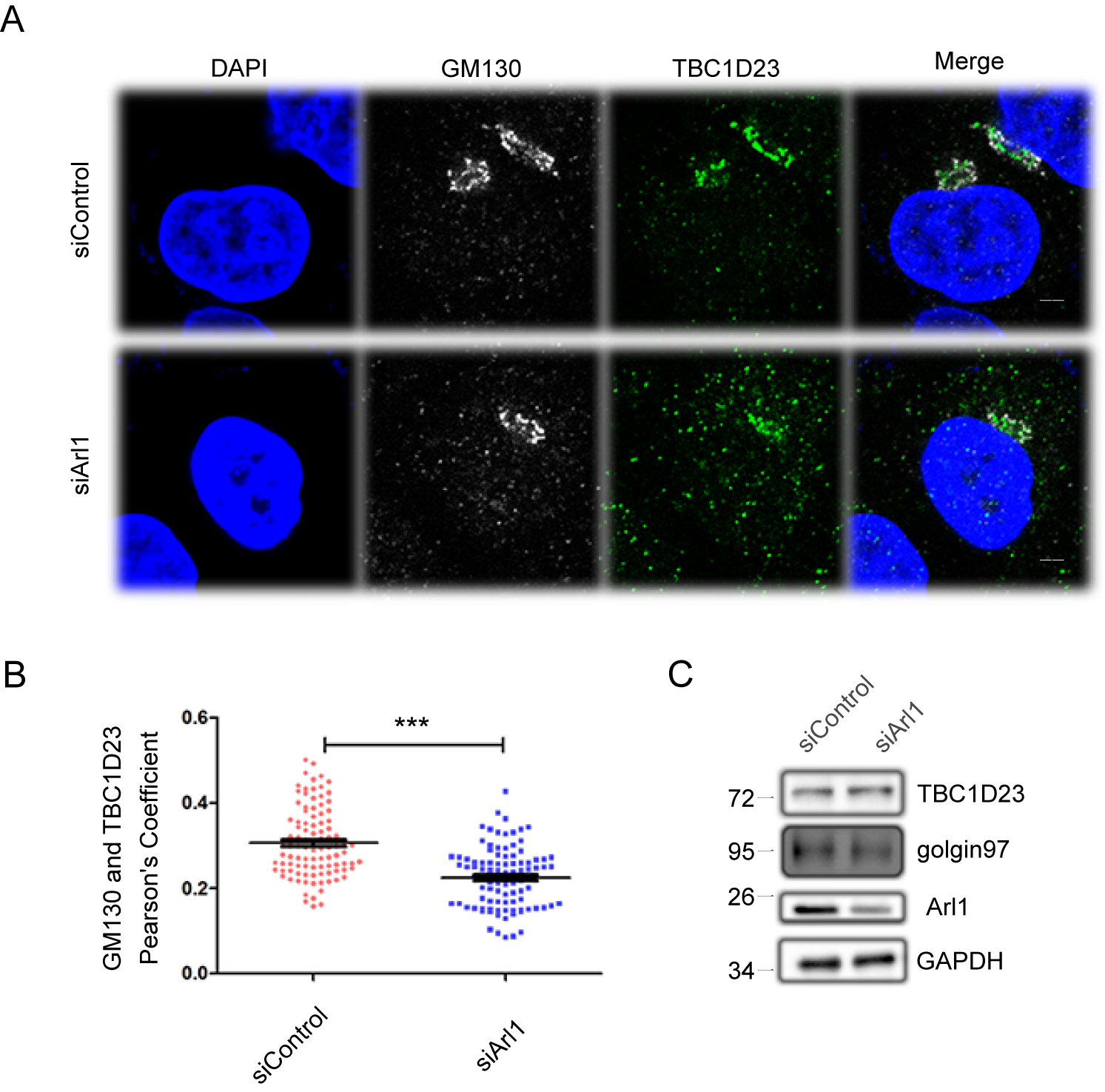

Supplement: S8 Fig — (A) Confocal immunofluorescence of HeLa cells transfected with siRNA targeting Arl1 or control siRNA. Cells were fixed 72 h after transfection and labeled with anti-CI-MPR (green) and GM130 (white) antibodies. Scale bar: 10 μm. (B) Quantitation of TBC1D23 colocalization with GM130 in cells as treated in (A); each point represents one cell. P values were calculated using unpaired t test. ***P < 0.0001. Experiments were triplicated, and the numerical data are included in S1 Data. (C) Immunoblot of whole-cell extracts for cells as treated in (A), showing the total protein levels of Arl1, golgin-97, and TBC1D23. CI-MPR, cation-independent mannose-6-phosphate receptor; siRNA, small interfering RNA. (TIF) [file pbio.3000746.s008.tif]

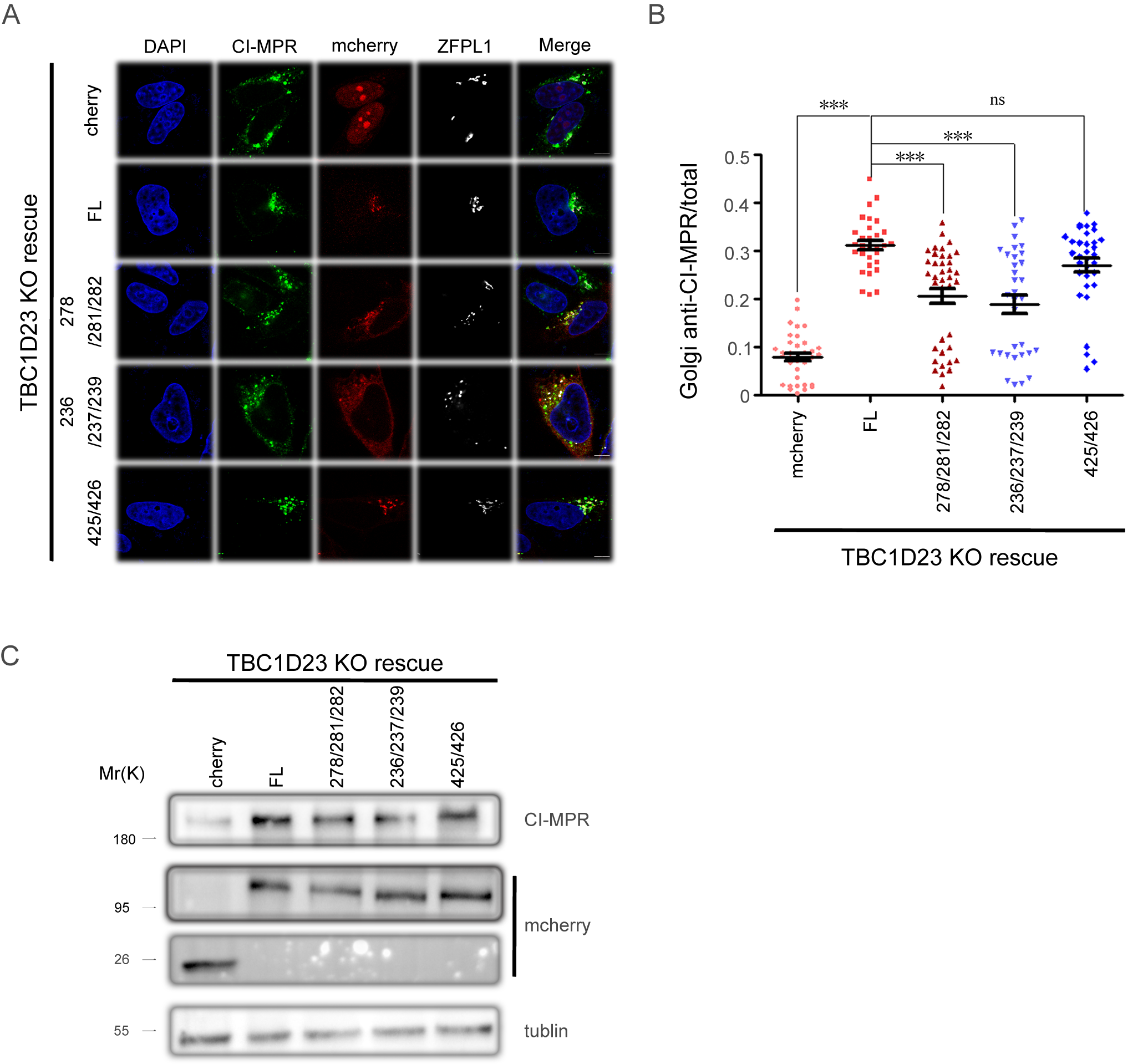

Supplement: S9 Fig — (A) Confocal immunofluorescence of TBC1D23 knockout HeLa cells transfected with vectors expressing mCherry, mCherry-TBC1D23-FL (“FL”), TBC1D23-L278A/Y281A/Y282A (“278/281/282”), TBC1D23-I236A/I237A/V239A (“236/237/239”), or TBC1D23-E425K/Y426A (“425/426”). The cells were fixed and labeled with anti-CI-MPR (green) and ZFPL1 (white) antibodies. Scale bar: 10 μm. (B) Quantitation of Golgi-localized CI-MPR over its total amount in cells treated as in (A). Each dot represents result from one cell. P values were calculated using one-way ANOVA, post hoc Tukey’s test. ***P < 0.0001. Experiments were triplicated, and the numerical data are included in S1 Data. (C) Immunoblot of whole-cell extracts of TBC1D23 knockout HeLa cells transfected with vectors expressing mCherry, mCherry-TBC1D23-FL (“FL”), TBC1D23-L278A/Y281A/Y282A (“278/281/282”), TBC1D23-I236A/I237A/V239A (“236/237/239”), or TBC1D23-E425K/Y426A (“425/426”). Cell lysates were probed with anti-cherry, TBC1D23, CI-MPR, or tubulin (control) antibodies. CI-MPR, cation-independent mannose-6-phosphate receptor; FL, full-length; ns, not significant; siRNA, small interfering RNA; ZFPL1, zinc finger protein like 1. (TIF) [file pbio.3000746.s009.tif]

Fig.3E

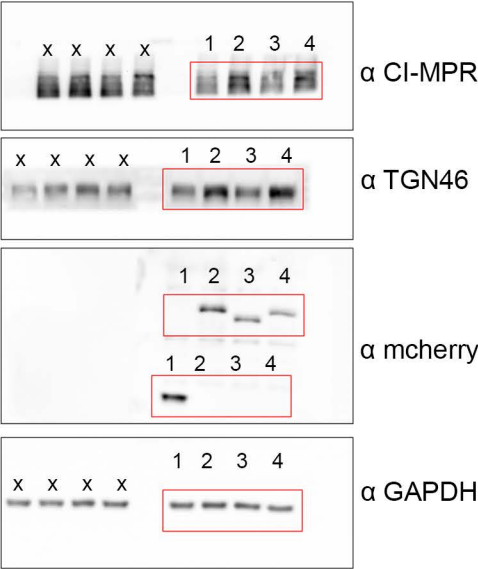

Fig.4C

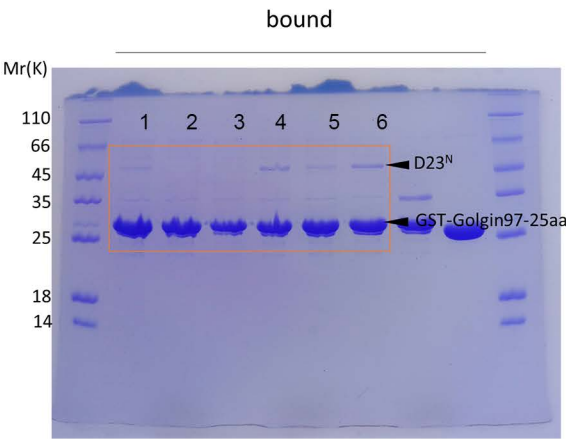

Fig.5A

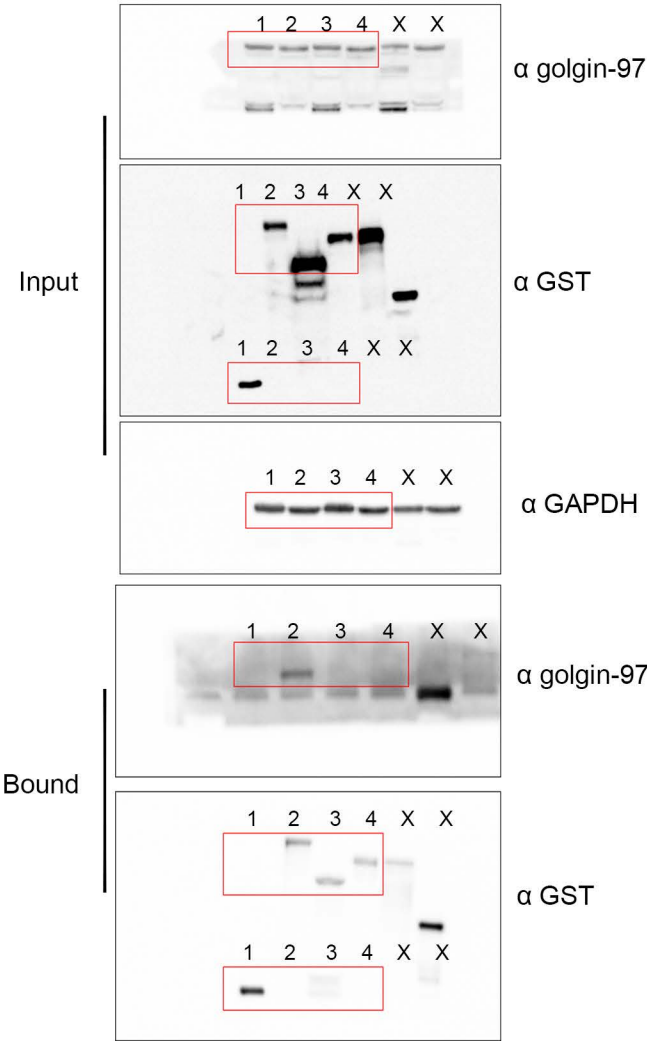

Fig.5B

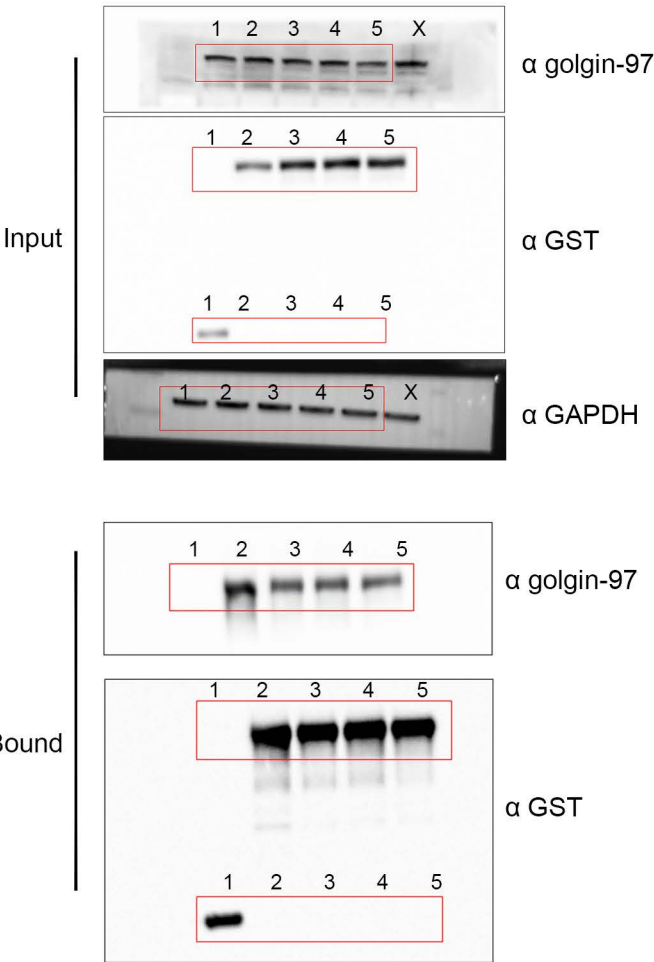

Fig.5E

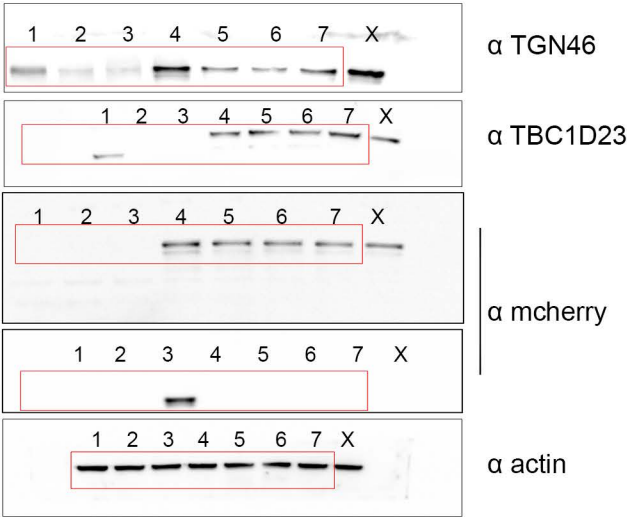

Fig.S6B

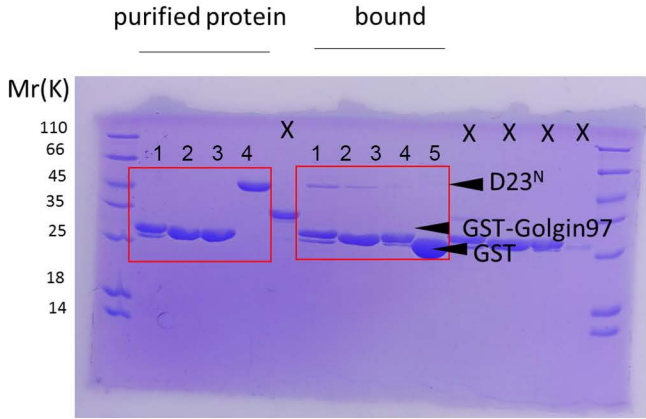

Fig.S6D

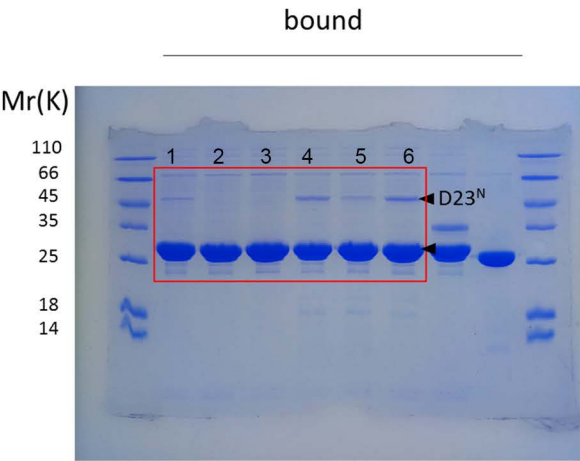

Fig.S6E

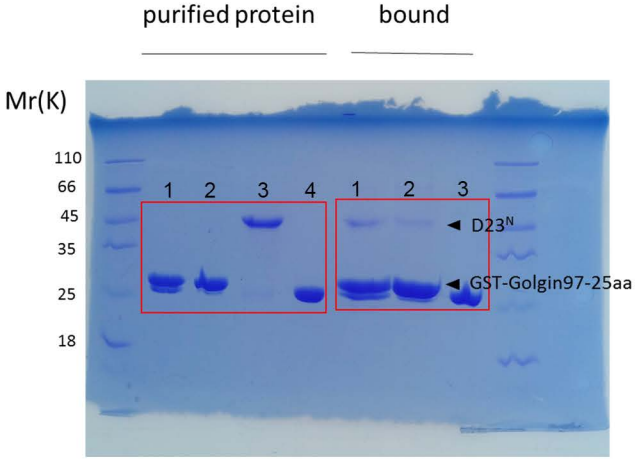

Fig.S6F

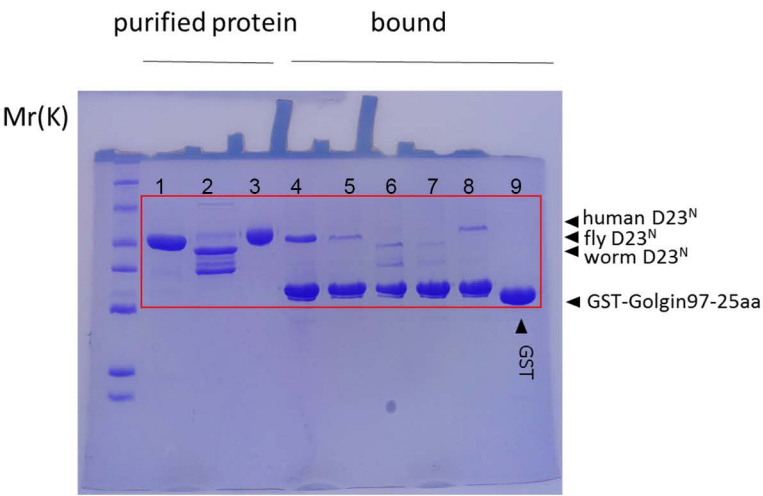

Fig.S7

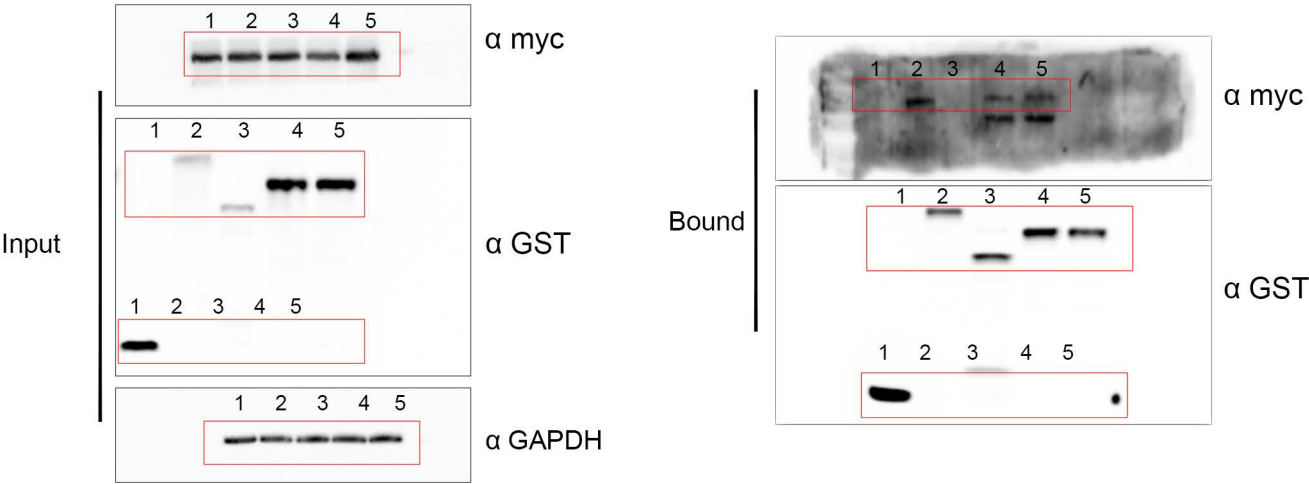

Fig.S8C

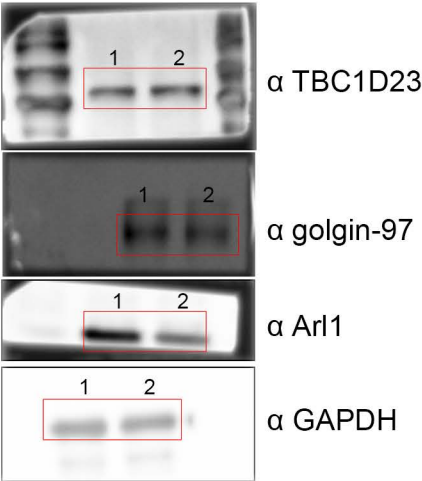

Fig.S9C

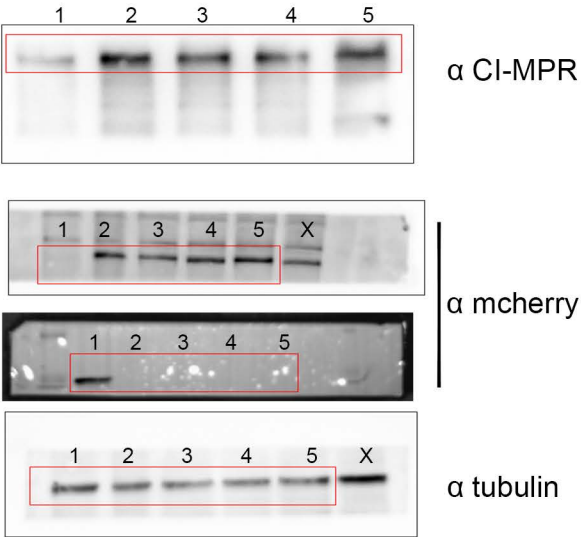

Supplement: S1 Raw images — (PDF) [file pbio.3000746.s014.pdf]
