# Supplementary material for: Structure of TBC1D23 N-terminus reveals a novel role for rhodanese domain
Source: PLoS Biol. 2020 May 26;18(5):e3000746. doi: 10.1371/journal.pbio.3000746 (PMC7274447; doi:10.1371/journal.pbio.3000746)
Supplement: S1 Table — (DOCX) [file pbio.3000746.s010.docx]

**S1 Table. Crystallography Data Collection and Refinement Statistics**

|  | TBC1D23^N^ (aa1-460) |
| --- | --- |
|  |  |
| Cell axial lengths (Å) | a=b=151.736 , c=38.604 |
| Spacegroup | P6_5_ |
|  |  |
| **Data collection** |  |
| Resolution range (Å) | 50.00-2.50 (2.54-2.50) |
| Number of observed reflections | 253183 (12642) |
| Number of unique reflections | 17980 (903) |
| Completeness (%) | 100.0 (100.0) |
| Redundancy | 14.1 (14.0) |
| R_pim_ | 0.051 (0.537) |
| Highest shell CC1/2 | 0.453 |
| Mean I/I_sigma_ | 6.2 (1.3) |
|  |  |
| **Refinement** |  |
| Resolution range (Å) | 49.72-2.50 (2.57-2.50) |
| Number of working reflections | 16040 (1251) |
| Number of test reflections | 955 (73) |
| R_work_^a^ (no. of reflections) | 0.217 (0.366) |
| R_free_^b^ (no. of reflections) | 0.252 (0.438) |
| R.m.s. deviation bond lengths (Å) | 0.003 |
| R.m.s. deviation bond angles (°) | 0.742 |
| Mean B value | 84.6 |
|  |  |
| **Ramachandran plot** |  |
| Most favored regions (%) | 92.8 |
| Allowed regions (%) | 7.2 |
| Disallowed regions (%) | 0.0 |

R_work_^a^ = Σ|Fo – Fc|/|Fo|, where Fc and Fo are the calculated and observed structure factor amplitudes, respectively
R_free_^b^ calculated as for R_work_ but for 5.0% of the total reflections chosen at random and omitted from refinement for all data sets

# values in the parenthesis is information from highest resolution shell.
