# Supplementary material for: Structure of TBC1D23 N-terminus reveals a novel role for rhodanese domain
Source: PLoS Biol. 2020 May 26;18(5):e3000746. doi: 10.1371/journal.pbio.3000746 (PMC7274447; doi:10.1371/journal.pbio.3000746)
Supplement: S2 Table — (DOCX) [file pbio.3000746.s011.docx]

**S2 Table. DNA Constructs Used in this Study.**

| **Construct name** | **Description** | **Source or reference** |
| --- | --- | --- |
| **TBC1D23** | | |
| **Bacterial expression** | | |
| TBC1D23 FL | GST-Tev-TBC1D23 (aa1-684) | (Huang et al, 2019) |
| TBC1D23^N^ | GST-TEV-TBC1D23 (aa1-460) | This study |
| TBC1D23^N^-236/237/239 | GST-TEV-TBC1D23 (aa1-460)-I236A/I237A/V239A | This study |
| TBC1D23^N^-278/281/282 | GST-TEV-TBC1D23 (aa1-460)- L278A/Y281A/Y282A | This study |
| TBC1D23^N^-179 | GST-TEV-TBC1D23 (aa1-460)-E179K | This study |
| TBC1D23^N^-396 | GST-TEV-TBC1D23 (aa1-460)-E396K | This study |
| TBC1D23^N^-425/426 | GST-TEV-TBC1D23 (aa1-460)-E425K/Y426A | This study |
| Fly TBC1D23^N^ | GST-TEV-FlyTBC1D23 (aa1-440) | This study |
| Worm TBC1D23^N^ | GST-TEV-WormTBC1D23 (aa1-421) | This study |
| Fly TBC1D23^N^ -I223A/I224A/I226A | GST-TEV-FlyTBC1D23 (aa1-440)-I223A/I224A/I226A | This study |
| Worm D23^N^-V220A/F221A/V223A | GST-TEV-WormTBC1D23 (aa1-421)-V220A/F221A/V223A | This study |
| **Eukaryotic expression** | | |
| GST-TBC1D23 FL | GST-TBC1D23 (aa1-684) | (Shin et al, 2017) |
| GST-TBC1D23 △TBC | GST-TBC1D23 (aa331-684) | This study |
| GST-TBC1D23 △Rhod1 | GST-TBC1D23 （aa1-330,514-685） | This study |
| GST-TBC1D23 FL-278/281/282 | GST-TBC1D23 FL-L278/Y281/Y282A | This study |
| GST-TBC1D23 FL-236/237/239 | GST-TBC1D23 FL-I236/I237/V239A | This study |
| GST-TBC1D23 FL-425/426 | GST-TBC1D23 FL-E425/Y426A | This study |
| mCherry- TBC1D23 FL | mCherry-N1- TBC1D23 FL | (Shin et al, 2017) |
| mCherry- TBC1D23 FL-278/281/282 | mCherry-N1- TBC1D23 FL-L278/Y281/Y282A | This study |
| mCherry- TBC1D23 FL-236/237/239 | mCherry-N1- TBC1D23 FL-I236/I237/V239A | This study |
| mCherry- TBC1D23 FL-425/426 | mCherry-N1- TBC1D23 FL-E425/Y426A | This study |
| mCherry- TBC1D23 △Rhod2 | mCherry-N1- TBC1D23 (aa1-330,461-684) | This study |
| mCherry- TBC1D23 FL-399/405 | mCherry-N1- TBC1D23 FL-C399S/R405A | This study |
| **Golgin-97/245** | | |
| Golgin-97-25aa | GST-TEV-Golgin-97 (aa1-25) | (Shin et al, 2017) |
| Golgin-97-17aa | GST-TEV-Golgin-97 (aa1-17) | This study |
| Golgin-97-12aa | GST-TEV-Golgin-97 (aa1-12) | This study |
| Golgin-97-8aa | GST-TEV-Golgin-97 (aa1-8) | This study |
| Golgin-245-25aa | GST-TEV-Golgin-245 (aa1-25) | (Shin et al, 2017) |
| Myc-Golgin-97 | Myc-Golgin-97-FL | This study |

| **Others** | | |
| --- | --- | --- |
| CDC25B | GST-Thrombin-HumanCDC25B (aa302-566) | (Reynolds et al,1999) |
| TSTD1 | His-TEV-TSTD1-FL | ( Libiad, et al, 2018) |
| CDC25B C473S | GST-Thrombin-HumanCDC25B (aa302-566)-C473S | (Reynolds et al,1999) |
| TSTD1 C79S | His-TEV-TSTD1-FLC79S | ( Libiad, et al, 2018) |
