# Supplementary material for: Structure of TBC1D23 N-terminus reveals a novel role for rhodanese domain
Source: PLoS Biol. 2020 May 26;18(5):e3000746. doi: 10.1371/journal.pbio.3000746 (PMC7274447; doi:10.1371/journal.pbio.3000746)
Supplement: S3 Table — (DOCX) [file pbio.3000746.s012.docx]

**S3 Table. Summary of Antibodies Used in this Study**

| Antibody | Company | Catalog | Concentration used or dilution fold |
| --- | --- | --- | --- |
| GST | Santa cruz | sc-138 | 1:1000(WB) |
| TGN46 | abcam | ab50595 | 1:300(IF)  1:1000(WB) |
| TBC1D23 | proteintech | 17002-1-AP | 1:1000(WB) |
| mCherry | proteintech | 26765-1-AP | 1:1500(WB) |
| Actin | proteintech | 66009-1-IG | 1:1000(WB) |
| tubulin | proteintech | 11224-1-AP | 1:1000(WB) |
| GAPDH | proteintech | 10494-1-AP | 1:1000(WB) |
| GM130 | BD | 610822 | 1:200(IF) |
| ZFLP1 | invitrogen | PA5-53254 | 1:250(IF) |
| CI-MPR | Bio-rad | Mca2048 | 1:200(IF) |
| Golgin-97 | absin | Abs122617 | 1:1000(WB) |
| Arl1 | proteintech | 16012-1-AP | 1:1000 |
| goat anti-mouse IgG-HRP | SAB | L3032-2 | 1:5000 |
| Goat anti-rabbit  IgG-HRP | SAB | L3012-2 | 1:5000 |
| FITC affinipure goat anti-mouse IgG | Jackson ImmunoResearch | 115-095-003 | 0.5 μg/ml |
| Goat anti-Rabbit IgG (H+L) Cross-Adsorbed Secondary Antibody, Alexa Fluor 546 | ThermoFisher | A-11010 | 1:2000 |
| Alexa Fluor 647 affinipure goat anti-mouse IgG | Jackson ImmunoResearch | 115-605-003 | 0.5 μg/ml |
| FITC affinipure goat anti-rabbit IgG | Jackson ImmunoResearch | 111-545-003 | 0.5 μg/ml |
