# Supplementary material for: Structure of TBC1D23 N-terminus reveals a novel role for rhodanese domain
Source: PLoS Biol. 2020 May 26;18(5):e3000746. doi: 10.1371/journal.pbio.3000746 (PMC7274447; doi:10.1371/journal.pbio.3000746)
Supplement: S4 Table — siRNA, short interfering RNA. (DOCX) [file pbio.3000746.s013.docx]

**S4 Table. Sequences of primers、Morpholino and siRNA**

| sqRT-PCR primer | |
| --- | --- |
| *Gapdh* | CTGGTGACCCGTGCTGCTTT (forward) |
|  | GTTTGCCGCCTTCTGCCTTA (reverse) |
| *Huc* | CTATGTGGATCCCAACGACGCCGAC (forward) |
|  | CAACTGCTCCATGTCTTTCTG (reverse) |
| Morpholino | |
| *Tbc1d23 MO1* | CTTCCCCTACAGCATCCGCCATTGC |
| *control* | CCTCTTACCTCAGTTACAATTTATA |
| siRNA | |
| *Arl1* | 5’-GAUUUAGGAGGACAGACAA-3’， 5’-CCAUACUGGAGAUGUUACU-3’  5’-CCGAAUUGGCAUUUCCAAA-3’, 5’-CGAAAAUGGCAGAUAUUCA-3’ |
| *control* | 5’-GGAGGCUGAACAUUCCGUC-3’ |
